# ﻿Morphology and molecular phylogeny reveal new species and records of Diaporthales from *Betula* in Xinjiang, China

**DOI:** 10.3897/mycokeys.125.169956

**Published:** 2025-11-18

**Authors:** Caixia Wang, Ning Jiang, Chuli Liu, ZiYan Xu, Hailong Lu, Rong Ma

**Affiliations:** 1 College of Forestry and Landscape Architecture, Xinjiang Agricultural University, Urumqi 830052, China Xinjiang Agricultural University Urumqi China; 2 Key Laboratory of Biodiversity Conservation of National Forestry and Grassland Administration, Ecology and Nature Conservation Institute, Chinese Academy of Forestry, Beijing 100091, China Chinese Academy of Forestry Beijing China; 3 The Key Laboratory for Silviculture and Conservation of the Ministry of Education, Beijing Forestry University, Beijing 100083, China Beijing Forestry University Beijing China; 4 Forestry Research Institute of Yili Kazakh Autonomous Prefecture, Yining 835099, Xinjiang, China Forestry Research Institute of Yili Kazakh Autonomous Prefecture Yining China

**Keywords:** *
Coryneum
*, *
Cryptosporella
*, *
Cytospora
*, *
Melanconis
*, molecular phylogeny, taxonomy

## Abstract

Xinjiang Uygur Autonomous Region (XUAR), located in the arid hinterland of northwest China, serves as important substrates for *Betula* species. As the most widely distributed floral genus of Betulaceae in Xinjiang, *Betula* serves as a vital pioneer species due to its stress tolerance and adaptation to extreme environments, playing an irreplaceable role in sustaining regional ecosystems. During disease surveys on *Betula* trees in the Tianshan and Altai Mountains of Xinjiang, we observed symptoms of stem and branch cankers. A total of 37 fungal strains were isolated and identified, based on morphological characteristics and phylogenetic analyses. As a result, *Cytospora altayensis***sp. nov.** was proposed and *Coryneum lanciforme*, *Cryptosporella betulae*, *Cr. tomentella*, *Cy. tanaitica* and *Melanconis groenlandica* are reported for the first time in China. In addition, *Cytospora sophoriopsis* is recorded for the first time on the host genus *Betula*. This study identifies potential pathogenic fungi associated with *Betula*, providing a foundation for future disease management and forest health research.

## ﻿Introduction

Xinjiang Uygur Autonomous Region (XUAR) is located in the central part of the Eurasian continent and features a typical temperate continental climate. Notably, its arid conditions foster unique biological species. Amongst these, *Betula* (birch trees) are an important plant genus in Xinjiang, primarily represented by *B. pendula* and *B. tianshanica*, which play significant ecological roles (Huang et al. 2020). As key pioneer species in the region, birch trees contribute greatly to natural forest conservation, ecological restoration, sustainable management and the improvement of the regional ecosystem (Dubois et al. 2020).

However, birch trees in the region are susceptible to various fungal pathogens. In particular, several fungal pathogens have been reported to cause branch canker, dieback and leaf spot diseases in birch species (Ivanová 2014; Fan et al. 2016a, b). For instance, *Melanconis stilbostoma* has been identified as a causative agent of birch cankers (Fan et al. 2016a, b), while species of *Diaporthe*, such as *D. betulae* and *D. betulicola*, have been associated with birch dieback (Du et al. 2016). Additionally, *Discula betulae* is known to cause birch leaf spot disease (Ivanová 2014). Despite these findings, systematic studies on these pathogens have not yet been conducted in XUAR.

Diaporthales comprise a diverse group of fungi that colonise various tree species, where they often act as pathogens infecting bark, leaves and fruits, though some species occur as endophytes or saprophytes (Rossman et al. 2007; Senanayake et al. 2017, 2018; Jiang et al. 2020a; Crous et al. 2023b; Xiao et al. 2023). Interestingly, members of this order exhibit a marked preference for hardwood hosts, particularly within families, such as Betulaceae, Fagaceae and Myrtaceae (Senanayake et al. 2017; Hyde et al. 2020b). Correspondingly, *Betula*, a genus including widespread tree species, has frequently been reported as a host associated with Diaporthales (Senanayake et al. 2017; Fan et al. 2018a; Crous et al. 2023a).

Taxa within Diaporthales can be clearly delineated using both morphological features and multi-gene phylogenetic analyses (Senanayake et al. 2017, 2018; Jiang et al. 2025b). On one hand, several genera possess distinctive traits: for instance, *Coryneum* species produce large, brown, distoseptate conidia (Jiang et al. 2018); *Cytospora* species are characterised by hyaline, allantoid ascospores and conidia (Fan et al. 2020); and *Asterosporium* species are recognised by their septate, brown, stellate conidia (Senanayake et al. 2017). On the other hand, some taxa, such as *Plagiostoma* and *Pseudoplagiostoma*, exhibit overlapping morphological features that complicate their differentiation (Senanayake et al. 2017; Jiang et al. 2025b, c).

In this study, samples exhibiting typical canker symptoms were collected from *Betula* hosts in XUAR. Fungal isolates were obtained and identified, based on morphological characteristics and molecular phylogenetic analyses, following contemporary taxonomic frameworks for the relevant taxa. The objectives of this study were to characterise species of Diaporthales associated with *Betula* hosts in XUAR and to establish links between these fungal species and their birch hosts to advance the understanding of canker diseases.

## ﻿Materials and methods

### ﻿Sample collection, isolation and morphology

During 2023 and 2024, we conducted branch canker disease surveys on hosts of *Betula* in XUAR. Barks exhibiting visible fungal fruiting bodies were collected for further analysis. All samples were stored in paper bags and transported to the laboratory for fungal isolation and morphological characterisation.

Fruiting bodies on branch samples were examined under a Zeiss Discovery V8 stereomicroscope (Jena, Germany), sectioned and transferred on to potato dextrose agar (PDA; containing 200 g potatoes, 20 g dextrose and 20 g agar per litre) plates at 25 °C to obtain fungal cultures. Specimens were deposited in the Herbarium of Xinjiang Agricultural University (XJAU), while pure cultures were preserved at the China Forestry Culture Collection Center (CFCC; https://cfcc.caf.ac.cn/).

Morphology was characterised mainly based on fruiting bodies forming on natural substrates. Stromata and conidiomata were hand-sectioned using a sterile double-edged blade and examined under a Zeiss Discovery V8 stereomicroscope. Microscopic structures including asci, ascospores, conidiophores, conidiogenous cells and conidia were observed and photographed using an Olympus BX51 compound microscope (Tokyo, Japan).

### ﻿Phylogenetic analyses

Genomic DNA was extracted from fungal colonies grown on PDA plates for two weeks using the Biospin Fungus Genomic DNA Extraction Kit-BSC14S1 (BioFlux, China), according to the manufacturer’s protocol. For preliminary fungal identification, the internal transcribed spacer (ITS) region was amplified using primer pairs ITS1/ITS4 (White et al. 1990). Further phylogenetic resolution was achieved by amplifying additional loci: the large subunit nrDNA (LSU) for *Coryneum* and *Melanconis* (primers LR0R/LR5); the partial actin (*act*) for *Cytospora* (primers ACT512F/ACT728R); the RNA polymerase II second largest subunit (*rpb2*) gene for *Coryneum*, *Cytospora* and *Melanconis* (primers RPB2-5F/fRPB2-7cR); the translation elongation factor 1-alpha (*tef1*) gene for *Coryneum*, *Cryptosporella*, *Cytospora* and *Melanconis* (primers EF1-728F/EF2; EF688F/1251R) and the partial beta-tubulin (*tub2*) gene for *Cryptosporella*, *Cytospora* and *Melanconis* (primers Bt2a/Bt2b) (Vilgalys and Hester 1990; Glass and Donaldson 1995; Carbone and Kohn 1999; Liu et al. 1999; Rehner 2001).

Polymerase chain reaction (PCR) amplification was performed under the following conditions: initial denaturation at 94 °C for 5 min; 35 cycles of denaturation (94 °C, 30 sec), annealing (48 °C for ITS/LSU, 54 °C for *tef1*/*tub2*, 55 °C for *rpb2* or 58 °C for *act*, 50 sec) and extension (72 °C, 1 min); followed by a final elongation at 72 °C for 7 min. The sequencing service was performed by Sangon Biotech Company Limited (Shanghai, China). Raw forward and reverse reads produced in this study were assembled and edited in Seqman v. 7.1.0 (DNASTAR Inc., USA) and deposited in the NCBI database (Tables 1–4). Multiple sequence alignments were generated using MAFFT v. 7 (Katoh and Standley 2013) and manually refined in MEGA v. 7.0.21.

**Table 1.** GenBank accession numbers used in the phylogenetic analyses of *Coryneum*.

Species	Strains	Hosts	GenBank accession numbers				References
			ITS	LSU	rpb2	tef1	
*Coryneum castaneicola*	CFCC 52315	*Castanea mollissima*	MH683559	MH683551	MH685723	MH685731	Jiang et al. (2018)
*Coryneum castaneicola*	CFCC 52316	*Castanea mollissima*	MH683560	MH683552	MH685724	MH685732	Jiang et al. (2018)
*Coryneum depressum*	D202	*Quercus petraea*	MH674330	MH674330	MH674334	MH674338	Jiang et al. (2018)
*Coryneum gigasporum*	CFCC 52319*	*Castanea mollissima*	MH683565	MH683557	MH685729	MH685737	Jiang et al. (2018)
*Coryneum gigasporum*	CFCC 52320	*Castanea mollissima*	MH683566	MH683558	MH685730	MH685738	Jiang et al. (2018)
*Coryneum gigasporum*	G14	*Castanea mollissima*	MK799957	MK799944	MK799820	MK799830	Jiang et al. (2019)
*Coryneum gigasporum*	G15	*Castanea mollissima*	MK799958	MK799945	MK799821	MK799831	Jiang et al. (2019)
*Coryneum heveanum*	MFLUCC 17-0369*	*Hevea brasiliensis*	MH778707	MH778703	NA	MH780881	Senwanna et al. (2018)
*Coryneum heveanum*	MFLUCC 17-0376	*Hevea brasiliensis*	MH778708	MH778704	NA	NA	Senwanna et al. (2018)
*Coryneum ilicis*	CFCC 52994*	*Ilex pernyi*	MK799948	MK799935	NA	NA	Jiang et al. (2019)
*Coryneum ilicis*	CFCC 52995	*Ilex pernyi*	MK799949	MK799936	NA	NA	Jiang et al. (2019)
*Coryneum ilicis*	CFCC 52996	*Ilex pernyi*	MK799950	MK799937	NA	NA	Jiang et al. (2019)
*Coryneum modonium*	D203	*Castanea sativa*	MH674331	MH674331	MH674335	MH674339	Jiang et al. (2018)
*Coryneum lanciforme*	D215	*Betula pubescens*	MH674332	MH674332	MH674336	MH674340	Jiang et al. (2018)
***Coryneum lanciforme***	**CFCC 71587**	***Betula pendula***	**PX227525**	**PX227507**	**PX233568**	**NA**	**In this study**
***Coryneum lanciforme***	**CFCC 71653**	***Betula pendula***	**PX227526**	**PX227508**	**PX233569**	**NA**	**In this study**
*Coryneum sinense*	CFCC 52452*	*Quercus serrata*	MH683561	MH683553	MH685725	MH685733	Jiang et al. (2018)
*Coryneum sinense*	CFCC 52453	*Quercus serrata*	MH683562	MH683554	MH685726	MH685734	Jiang et al. (2018)
*Coryneum sinense*	X23	*Quercus serrata*	MK799952	MK799939	MK799816	MK799826	Jiang et al. (2019)
*Coryneum sinense*	X60	*Quercus serrata*	MK799953	MK799940	MK799814	MK799824	Jiang et al. (2019)
*Coryneum songshanense*	CFCC 52997*	*Quercus dentata*	MK799946	MK799933	MK799812	MK799822	Jiang et al. (2019)
*Coryneum songshanense*	CFCC 52998	*Quercus dentata*	MK799947	MK799934	MK799813	MK799823	Jiang et al. (2019)
*Coryneum suttonii*	CFCC 52317*	*Castanea mollissima*	MH683563	MH683555	MH685727	MH685735	Jiang et al. (2018)
*Coryneum suttonii*	CFCC 52318	*Castanea mollissima*	MH683564	MH683556	MH685728	MH685736	Jiang et al. (2018)
*Coryneum suttonii*	Z17	*Castanea mollissima*	MK799955	MK799942	MK799818	MK799828	Jiang et al. (2019)
*Coryneum suttonii*	Z86	*Castanea mollissima*	MK799956	MK799943	MK799819	MK799829	Jiang et al. (2019)
*Coryneum umbonatum*	D201	*Quercus robur*	MH674329	MH674329	MH674333	MH674337	Jiang et al. (2018)
*Coryneum fagi*	BJFC-S1782*	*Fagus* sp.	MW144761	MW144953	NA	NA	Boonmee et al. (2021)
*Coryneum fagi*	BJFC-S1783	*Fagus* sp.	MW144762	MW144954	NA	NA	Boonmee et al. (2021)
*Coryneum septemseptatum*	GMB0393	decaying wood	OQ540748	OQ540743	NA	NA	Long et al. (2023)
*Coryneum septemseptatum*	GMB0392*	decaying wood	OQ560328	OQ560329	NA	NA	Long et al. (2023)
*Hyaloterminalis alishanensis*	NCYUCC 19-0400*	*Cerasus* sp.	MT447559	MT447557	NA	NA	Rathnayaka et al. (2020)

**Note**. “NA” indicates unavailable sequences, sequences produced in the current study are in bold and * means ex-type strains.

**Table 2.** GenBank accession numbers used in the phylogenetic analyses of *Cytospora*.

Species	Strains	GenBank accession numbers					References
		ITS	act	rpb2	tef1	tub2	
*Cytospora ailanthicola*	CFCC 54064	PP988711	NA	PQ074892	PQ074251	PQ075209	Lin et al. (2024)
*Cytospora ailanthicola*	CFCC 89970*	MH933618	MH933526	MH933592	MH933494	MH933565	Fan et al. (2020)
***Cytospora altayensis* sp. nov.**	**CFCC 71686**	**PX227527**	**PX233570**	**PX233584**	**PX233598**	**PX233612**	**In this study**
***Cytospora altayensis* sp. nov.**	**CFCC 71687**	**PX227528**	**PX233571**	**PX233585**	**PX233599**	**PX233613**	**In this study**
***Cytospora altayensis* sp. nov.**	**CFCC 71688**	**PX227529**	**PX233572**	**PX233586**	**PX233600**	**PX233614**	**In this study**
*Cytospora atrocirrhata*	CFCC 59056	PP988739	PQ074599	PQ074912	PQ074274	PQ075229	Lin et al. (2024)
*Cytospora atrocirrhata*	CFCC 89615	KR045618	KF498673	KU710946	KP310858	KR045659	Fan et al. (2015)
*Cytospora auerswaldii*	CBS 153.29	PP988740	PQ074600	PQ074913	PQ074275	PQ075230	Lin et al. (2024)
*Cytospora berberidis*	CFCC 89927*	KR045620	KU710990	KU710948	KU710913	KR045661	Liu et al. (2015)
*Cytospora berberidis*	CFCC 89933	KR045621	KU710991	KU710949	KU710914	KR045662	Liu et al. (2015)
*Cytospora betulae*	CBS 141622*	PP988752	PQ074610	PQ074922	PQ074284	PQ075236	Lin et al. (2024)
*Cytospora celtidicola*	CFCC 50497*	MH933623	NA	MH933595	MH933499	MH933566	Fan et al. (2020)
*Cytospora chrysosperma*	CBS 120.83	PP988773	PQ074626	PQ074942	PQ074304	PQ075255	Lin et al. (2024)
*Cytospora chrysosperma*	CBS 197.50*	PP988777	PQ074630	PQ074946	PQ074308	PQ075259	Lin et al. (2024)
*Cytospora eastringensis*	CFCC 58222*	PP988818	NA	PQ074980	PQ074346	NA	Lin et al. (2024)
*Cytospora guyuanensis*	CFCC 55855*	PP988853	NA	PQ075011	PQ074378	PQ075323	Lin et al. (2024)
*Cytospora hejingensis*	CFCC 59571*	PP060455	PP059657	PP059663	PP059667	PP059673	Wang et al. (2024)
*Cytospora hippophaopsis*	CGMCC 3.18997*	PP965505	PP957863	NA	PP957877	PP957884	Cai et al. (2024)
*Cytospora iranica*	IRAN 4200C*	MW295652	MZ014512	MW824359	MW394146	NA	Hanifeh et al. (2022)
*Cytospora jiufengensis*	CFCC 55839*	PP988865	PQ074698	NA	NA	PQ075332	Lin et al. (2024)
*Cytospora joaquinensis*	CBS 144235	MG971895	MG972044	NA	MG971605	NA	Lawrence et al. (2018)
*Cytospora kuanchengensis*	CFCC 52464*	MK432616	MK442940	MK578076	NA	NA	Jiang et al. (2020b)
*Cytospora leucosperma*	CBS 109491	PP988884	PQ074714	PQ075035	PQ074407	PQ075347	Lin et al. (2024)
*Cytospora longispora*	CBS 144236*	MG971905	MG972054	NA	MG971615	NA	Lawrence et al. (2018)
*Cytospora longistiolata*	MFLUCC 16-0628	KY417734	KY417700	KY417802	NA	NA	Norphanphoun et al. (2017)
*Cytospora macropycnidia*	CBS 149338*	OP038094	OP003977	OP095265	OP106954	OP079909	Travadon et al. (2022)
*Cytospora melnikii*	CFCC 89984	MH933644	MH933551	MH933609	MH933515	MH933580	Pan et al. (2018)
*Cytospora nobilis*	CFCC 58227	PP988928	PQ074756	PQ075075	PQ074449	PQ075389	Lin et al. (2024)
*Cytospora nobilis*	CFCC 58228	PP988929	PQ074757	PQ075076	PQ074450	PQ075390	Lin et al. (2024)
*Cytospora oleicola*	CBS 144248*	MG971944	MG972098	NA	MG971660	NA	Lawrence et al. (2018)
*Cytospora platycladicola*	CFCC 50038*	KT222840	MH933555	MH933613	MH933519	MH933584	Yang et al. (2015)
*Cytospora populinopsis*	CFCC 50032*	MH933648	MH933556	MH933614	MH933520	MH933585	Fan et al. (2020)
*Cytospora pruinopsis*	CFCC 50034*	KP281259	KP310836	KU710970	KP310849	KP310819	Yang et al. (2015)
*Cytospora pruinopsis*	CFCC 50035	KP281260	KP310837	KU710971	KP310850	KP310820	Yang et al. (2015)
*Cytospora pruinosa*	CBS 200.42	PP988951	PQ074777	PQ075095	PQ074470	PQ075410	Lin et al. (2024)
*Cytospora prunicola*	MFLU 17-0995*	MG742350	MG742353	MG742352	NA	NA	Hyde et al. (2018)
*Cytospora pseudochrysosperma*	CFCC 54081*	MZ702631	NA	NA	OK303613	OK303680	Lin et al. (2024)
*Cytospora pseudochrysosperma*	CFCC 89629	KF765673	NA	KF765705	NA	NA	Lin et al. (2024)
*Cytospora pseudochrysosperma*	CFCC 89981*	MH933625	MH933533	MH933597	MH933501	MH933568	Lin et al. (2024)
*Cytospora qinghaiensis*	CFCC 50026*	KP281267	KP310843	KU710972	KP310856	KP310826	Yang et al. (2015)
*Cytospora ribis*	CBS 187.36	PP988963	PQ074788	PQ075106	PQ074480	PQ075420	Lin et al. (2024)
*Cytospora rosigena*	MFLUCC 18-0921*	MN879872	NA	NA	NA	NA	Hyde et al. (2020a)
*Cytospora rostrata*	CFCC 89909*	KR045643	KU711009	KU710974	NA	NA	Fan et al. (2020)
*Cytospora rostrata*	CFCC 89910	KR045644	KU711010	KU710975	KU710933	NA	Fan et al. (2020)
*Cytospora salicacearum*	MFLUCC 15-0509*	KY417746	KY417712	KY417814	NA	NA	Norphanphoun et al. (2017)
*Cytospora salicina*	CBS 507.77	PP988981	PQ074804	PQ075122	PQ074497	PQ075435	Lin et al. (2024)
*Cytospora salicina*	MFLUCC 15-0862*	KY417750	KY417716	KY417818	NA	NA	Norphanphoun et al. (2017)
*Cytospora shaanxiensis*	CFCC 56032*	PP988987	PQ074810	PQ075128	PQ074502	PQ075441	Lin et al. (2024)
*Cytospora sibiraeae*	CFCC 50045*	KR045651	KU711015	KU710982	KU710938	KR045692	Liu et al. (2015)
*Cytospora sibiraeae*	CFCC 50046	KR045652	KU711015	KU710983	KU710939	KR045693	Liu et al. (2015)
*Cytospora sidaohensis*	CFCC 56042*	PP988992	PQ074815	PQ075133	PQ074507	PQ075446	Lin et al. (2024)
*Cytospora sinensis*	CFCC 58231	PP988995	PQ074818	PQ075136	PQ074510	PQ075449	Lin et al. (2024)
*Cytospora sinensis*	CFCC 58235*	PP988997	PQ074820	PQ075138	PQ074512	PQ075451	Lin et al. (2024)
*Cytospora sinensis*	CFCC 58471	PP989002	PQ074824	PQ075143	PQ074517	PQ075455	Lin et al. (2024)
*Cytospora songshanensis*	CFCC 56351*	PP989006	PQ074828	PQ075147	PQ074521	PQ075459	Lin et al. (2024)
*Cytospora sophoriopsis*	CFCC 58464	PP989009	PQ074831	PQ075150	PQ074524	PQ075461	Lin et al. (2024)
*Cytospora sophoriopsis*	CFCC 89600*	KR045623	KU710992	KU710951	KU710915	KP310817	Fan et al. (2020)
***Cytospora sophoriopsis***	**CFCC 71679**	**PX227530**	**PX233573**	**PX233587**	**PX233601**	**PX233615**	**In this study**
***Cytospora sophoriopsis***	**CFCC 71680**	**PX227531**	**PX233574**	**PX233588**	**PX233602**	**PX233616**	**In this study**
***Cytospora sophoriopsis***	**CFCC 71681**	**PX227532**	**PX233575**	**PX233589**	**PX233603**	**PX233617**	**In this study**
***Cytospora sophoriopsis***	**CFCC 71682**	**PX227533**	**PX233576**	**PX233590**	**PX233604**	**PX233618**	**In this study**
*Cytospora syringina*	CFCC 50036*	KP310800	KP310832	NA	KP310845	KP310815	Lin et al. (2024)
*Cytospora tanaitica*	MFLUCC 14-1057*	KT459411	KT459413	NA	NA	NA	Ariyawansa et al. (2015)
***Cytospora tanaitic***a	**CFCC 71675**	**PX227534**	**PX233577**	**PX233591**	**PX233605**	**PX233637**	**In this study**
***Cytospora tanaitica***	**CFCC 71676**	**PX227535**	**PX233578**	**PX233592**	**PX233606**	**PX233638**	**In this study**
***Cytospora tanaitica***	**CFCC 71677**	**PX227536**	**PX233579**	**PX233593**	**PX233607**	**PX233639**	**In this study**
***Cytospora tanaitica***	**CFCC 71678**	**PX227537**	**PX233580**	**PX233594**	**PX233608**	**PX233640**	**In this study**
***Cytospora tanaitic***a	**CFCC 71683**	**PX227538**	**PX233581**	**PX233595**	**PX233609**	**PX233641**	**In this study**
***Cytospora tanaitica***	**CFCC 71684**	**PX227539**	**PX233582**	**PX233596**	**PX233610**	**PX233642**	**In this study**
***Cytospora tanaitica***	**CFCC 71685**	**PX227540**	**PX233583**	**PX233597**	**PX233611**	**PX233643**	**In this study**
*Cytospora tenebrica*	CFCC 55841*	PP989019	PQ074838	PQ075160	PQ074534	PQ075471	Lin et al. (2024)
*Cytospora tetraspora*	CFCC 55847*	PP989021	PQ074840	PQ075162	PQ074536	PQ075473	Lin et al. (2024)
*Cytospora tetraspora*	CFCC 56279*	PP989023	PQ074842	PQ075164	PQ074538	PQ075475	Lin et al. (2024)
*Cytospora tritici*	CBS 118561	PP989038	PQ074856	PQ075175	PQ074549	PQ075486	Lin et al. (2024)
*Cytospora tritici*	CBS 118563	PP989039	PQ074857	PQ075176	PQ074550	PQ075487	Lin et al. (2024)
*Cytospora ulmi*	MFLUCC 15-0863*	KY417759	KY417725	KY417827	NA	NA	Norphanphoun et al. (2017)
*Cytospora ulmicola*	MFLUCC 18-1227*	MH940220	MH940216	NA	NA	NA	Phookamsak et al. (2019)
*Cytospora uniloculata*	CFCC 58460*	PP989043	PQ074861	NA	PQ074554	NA	Lin et al. (2024)
*Cytospora washingtonensis*	CBS 141619*	PP989065	PQ074874	PQ075192	PQ074569	PQ075502	Lin et al. (2024)
*Cytospora xiaolongmenensis*	CFCC 58459*	PP989068	PQ074877	PQ075194	PQ074572	PQ075505	Lin et al. (2024)
*Cytospora yakimana*	CBS 149297*	OM976602	ON012555	ON045093	ON012569	ON086750	Travadon et al. (2022)
*Cytospora yuduensis*	CFCC 57539*	PP989070	PQ074879	PQ075196	PQ074574	PQ075507	Lin et al. (2024)
*Diaporthe vaccinii*	CBS 160.32	KC343228	JQ807297	NA	KC343954	KC344196	Gomes et al. (2013)

**Note**. “NA” indicates unavailable sequences, sequences produced in the current study are in bold and * means ex-type strains.

**Table 3.** GenBank accession numbers used in the phylogenetic analyses of *Cryptosporella*.

Species	Strains	Hosts	GenBank accession numbers			References
			ITS	tef1	tub2	
*Cryptosporella alnicola*	CBS 121074	*Corylus cornuta*	EU199204	EU221960	EU219138	Mejía et al. (2008)
*Cryptosporella alni-rubrae*	CBS 126120	*Alnus rubra*	GU826092	GU826051	GU826010	Mejía et al. (2011)
*Cryptosporella alni-rubrae*	LCM411	*Alnus rubra*	GU826090	GU826049	GU826008	Mejía et al. (2011)
*Cryptosporella alni-rubrae*	LCM499.01*	*Alnus rubra*	GU826096	GU826055	GU826014	Mejía et al. (2011)
*Cryptosporella alni-sinuatae*	AR4200	Alnus viridis ssp. sinuata	GU826086	GU826045	GU825989	Mejía et al. (2011)
*Cryptosporella alni-sinuatae*	CBS 125662*	Alnus viridis ssp. sinuata	GU826087	GU826046	GU826005	Mejía et al. (2011)
*Cryptosporella alni-tenuifoliae*	CBS 125663*	Alnus incana ssp. tenuifol	GU826097	GU826056	GU826015	Mejía et al. (2011)
*Cryptosporella alni-cordatae*	MFLUCC 16-0485	*Alnus* sp.	KY797640	NA	NA	Tian et al. (2018)
*Cryptosporella alni-cordatae*	MFLU 16-0812	*Alnus* sp.	KY797639	NA	NA	Tian et al. (2018)
*Cryptosporella amistadensis*	LCM618.01	*Alnus acuminata*	GU826109	GU826073	GU826032	Mejía et al. (2011)
*Cryptosporella amistadensis*	CBS 125664*	*Alnus acuminata*	GU826108	GU826072	GU826031	Mejía et al. (2011)
*Cryptosporella betulae*	CBS 121078	*Betula pendula*	EU199213	GU826057	GU826016	Mejía et al. (2008)
*Cryptosporella betulae*	LCM477.01	*Betula pendula*	GU826098	GU826059	GU826018	Mejía et al. (2011)
*Cryptosporella betulae*	CBS 121079	*Betula pendula*	EU199216	GU826058	GU826017	Mejía et al. (2008)
***Cryptosporella betulae***	**CFCC 71654**	***Betula pendula***	**PX227541**	**PX233644**	**PX233656**	**In this study**
***Cryptosporella betulae***	**CFCC 71655**	***Betula pendula***	**PX227542**	**PX233645**	**PX233657**	**In this study**
***Cryptosporella betulae***	**CFCC 71656**	***Betula pendula***	**PX227543**	**PX233646**	**PX233658**	**In this study**
***Cryptosporella betulae***	**CFCC 71657**	***Betula pendula***	**PX227544**	**PX233647**	**PX233659**	**In this study**
***Cryptosporella betulae***	**CFCC 71658**	***Betula pendula***	**PX227545**	**PX233648**	**PX233660**	**In this study**
*Cryptosporella confusa*	CBS 121003	*Betula papyrifera*	EU199219	NA	NA	Mejía et al. (2008)
*Cryptosporella corylina*	LCM391.04	*Corylus avellana*	GU826100	GU826063	GU826022	Mejía et al. (2011)
*Cryptosporella femoralis*	CBS 121076*	Alnus incana ssp. rugosa	EU199220	EU221951	EU219139	Mejía et al. (2008)
*Cryptosporella femoralis*	LCM196.04	Alnus incana ssp. rugosa	GU826102	GU826067	GU826025	Mejía et al. (2011)
*Cryptosporella hypodermia*	CBS 109753	*Ulmus minor*	EU199224	GU826064	GU826023	Mejía et al. (2008)
*Cryptosporella hypodermia*	CBS 122593*	*Ulmus minor*	EU199181	GU826066	GU826024	Mejía et al. (2008)
*Cryptosporella jaklitschii*	LCM112.01	*Alnus serrulata*	GU826089	GU826048	GU826007	Mejía et al. (2011)
*Cryptosporella jaklitschii*	CBS 125665*	*Alnus serrulata*	GU826088	GU826047	GU826006	Mejía et al. (2011)
*Cryptosporella marylandica*	LCM386.05	*Alnus maritima*	GU826106	GU826070	GU826029	Mejía et al. (2011)
*Cryptosporella marylandica*	LCM581.01	*Alnus maritima*	GU826107	GU826071	GU826030	Mejía et al. (2011)
*Cryptosporella marylandica*	CBS 125666*	*Alnus maritima*	GU826105	GU826069	GU826028	Mejía et al. (2011)
*Cryptosporella multicontinentalis*	CBS 126119	Alnus incana ssp. rugosa	GU826081	GU826040	GU825999	Mejía et al. (2011)
*Cryptosporella multicontinentalis*	LCM93b.02	Alnus incana ssp. rugosa	GU826082	GU826041	GU826000	Mejía et al. (2011)
*Cryptosporella multicontinentalis*	LCM427.01	*Alnus glutinosa*	GU826085	GU826044	GU826004	Mejía et al. (2011)
*Cryptosporella multicontinentalis*	LCM401.01*	*Alnus glutinosa*	GU826083	GU826042	GU826001	Mejía et al. (2011)
*Cryptosporella pacifica*	LCM453.01	Alnus incana ssp. tenuifolia	GU826077	GU826037	GU825995	Mejía et al. (2011)
*Cryptosporella pacifica*	LCM461.01*	Alnus incana ssp. tenuifolia	GU826076	GU826036	GU825994	Mejía et al. (2011)
*Cryptosporella platyphylla*	CFCC 50466	*Betula platyphylla*	KT732947	KT733015	KT733019	Fan et al. (2016b)
*Cryptosporella platyphylla*	CFCC 50465*	*Betula platyphylla*	KT732946	KT733014	KT733018	Fan et al. (2016b)
*Cryptosporella suffusa*	LCM576.01	*Alnus* sp.	GU826079	GU826039	GU825997	Mejía et al. (2011)
*Cryptosporella suffusa*	LCM576.03	*Alnus* sp.	GU826078	GU826038	GU825996	Mejía et al. (2011)
*Cryptosporella tomentella*	CBS 126440	*Betula alleghaniensis*	GU826099	GU826062	GU826021	Mejía et al. (2011)
*Cryptosporella tomentella*	CBS 121073	*Betula* sp.	EU199217	GU826060	GU826019	Mejía et al. (2008)
***Cryptosporella tomentella***	**CFCC 71659**	***Betula microphylla***	**PX227546**	**PX233649**	**PX233661**	**In this study**
***Cryptosporella tomentella***	**CFCC 71660**	***Betula microphylla***	**PX227547**	**PX233650**	**PX233662**	**In this study**
***Cryptosporella tomentella***	**CFCC 71661**	***Betula microphylla***	**PX227548**	**PX233651**	**PX233663**	**In this study**
***Cryptosporella tomentella***	**CFCC 71662**	***Betula microphylla***	**PX227549**	**PX233652**	**PX233664**	**In this study**
***Cryptosporella tomentella***	**CFCC 71663**	***Betula microphylla***	**PX227550**	**PX233653**	**PX233665**	**In this study**
***Cryptosporella tomentella***	**CFCC 71664**	***Betula microphylla***	**PX227551**	**PX233654**	**PX233666**	**In this study**
***Cryptosporella tomentella***	**CFCC 71665**	***Betula microphylla***	**PX227552**	**PX233655**	**PX233667**	**In this study**
*Cryptosporella wehmeyeriana*	LCM85.02	*Tilia americana*	GU826104	GU826068	GU826027	Mejía et al. (2011)
*Ditopella ditopa*	LCM94.02	Alnus incana ssp. rugosa	GU826075	GU826033	GU825990	Mejía et al. (2011)

**Note**. “NA” indicates unavailable sequences, sequences produced in the current study are in bold and * means ex-type strains.

**Table 4.** GenBank accession numbers used in the phylogenetic analyses of *Melanconis*.

Taxa	Strains	Hosts	GenBank accession numbers					References
			ITS	LSU	rpb2	tef1	tub2	
*Juglanconis juglandina*	CBS 133343	*Juglans regia*	KY427149	KY427149	KY427199	KY427218	KY427234	Voglmayr et al. (2017)
*Juglanconis pterocaryae*	CBS 144326*	*Pterocarya fraxinifolia*	MK229175	MK229175	MK238324	MK238332	MK238338	Voglmayr et al. (2017)
*Melanconis alni*	CBS 131693	*Alnus glutinosa*	MN784962	MN784962	MN780745	MN780774	MN780803	Jaklitsch and Voglmayr (2020)
*Melanconis alni*	CBS 131695*	*Alnus glutinosa*	MN784963	MN784963	MN780746	MN780775	MN780804	Jaklitsch and Voglmayr (2020)
*Melanconis alni*	MEW*	*Alnus glutinosa*	MN784964	MN784964	MN780747	MN780776	MN780805	Jaklitsch and Voglmayr (2020)
*Melanconis alni*	MAIV	*Alnus incana*	MN784965	MN784965	MN780748	MN780777	MN780806	Jaklitsch and Voglmayr (2020)
*Melanconis alni*	D156	*Alnus glutinosa*	MN784966	MN784966	MN780749	MN780778	MN780807	Jaklitsch and Voglmayr (2020)
*Melanconis betulae*	CFCC 50471*	*Betula albosinensis*	KT732952	KT732971	KT732984	KT733001	KT733022	Fan et al. (2016a)
*Melanconis betulae*	CFCC 50472	*Betula albosinensis*	KT732953	KT732972	KT732985	KT733002	KT733023	Fan et al. (2016a)
*Melanconis betulae*	CFCC 50473	*Betula albosinensis*	KT732954	KT732973	KT732986	KT733003	KT733024	Fan et al. (2016a)
*Melanconis groenlandica*	CBS 116450*	*Betula nana*	KU878552	KU878553	NA	KU878554	KU878555	Lombard et al. (2016)
*Melanconis groenlandica*	MAFF 410219	*Betula maximowicziana*	MN784967	MN784967	MN780750	MN780779	MN780808	Jaklitsch and Voglmayr (2020)
*Melanconis groenlandica*	CBS 133341	*Betula papyrifera*	MN784968	MN784968	MN780751	MN780780	MN780809	Jaklitsch and Voglmayr (2020)
*Melanconis groenlandica*	CBS 133339	*Betula* sp.	MN784969	MN784969	MN780752	MN780781	MN780810	Jaklitsch and Voglmayr (2020)
*Melanconis groenlandica*	CBS 133340	*Betula papyrifera*	MN784970	MN784970	MN780753	MN780782	MN780811	Jaklitsch and Voglmayr (2020)
***Melanconis groenlandica***	**CFCC 71566**	***Betula tianchanica***	**PX227553**	**PX227509**	**NA**	**PX233619**	**PX233628**	**In this study**
***Melanconis groenlandica***	**CFCC 71567**	***Betula tianchanica***	**PX227554**	**PX227510**	**NA**	**PX233620**	**PX233629**	**In this study**
***Melanconis groenlandica***	**CFCC 71568**	***Betula tianchanica***	**PX227555**	**PX227511**	**NA**	**PX233621**	**PX233630**	**In this study**
***Melanconis groenlandica***	**CFCC 71569**	***Betula tianchanica***	**PX227556**	**PX227512**	**NA**	**PX233622**	**PX233631**	**In this study**
*Melanconis itoana*	MAFF 410080	*Betula ermanii*	MN784971	MN784971	MN780754	MN780783	MN780812	Jaklitsch and Voglmayr (2020)
*Melanconis itoana*	CFCC 50474	*Betula albosinensis*	KT732955	KT732974	KT732987	KT733004	KT733025	Fan et al. (2016a)
*Melanconis itoana*	CFCC 52876	*Betula albosinensis*	MK096324	MK096364	MK096409	MK096284	NA	Fan et al. (2016a)
*Melanconis itoana*	CFCC 52877	*Betula albosinensis*	MK096326	MK096366	MK096411	MK096286	NA	Fan et al. (2016a)
*Melanconis itoana*	CFCC 52878	*Betula albosinensis*	MK096327	MK096367	MK096412	MK096287	NA	Fan et al. (2016a)
*Melanconis larissae*	CBS 123196*	*Betula* sp.	MN784972	MN784972	MN780755	MN780784	MN780813	Jaklitsch and Voglmayr (2020)
Melanconis marginalis subsp. europaea	D157	*Alnus alnobetula*	MN784973	MN784973	MN780756	MN780785	NA	Jaklitsch and Voglmayr (2020)
Melanconis marginalis subsp. europaea	D158	*Alnus alnobetula*	MN784974	MN784974	MN780757	MN780786	MN780814	Jaklitsch and Voglmayr (2020)
Melanconis marginalis subsp. europaea	D257	*Alnus incana*	MN784975	MN784975	MN780758	MN780787	MN780815	Jaklitsch and Voglmayr (2020)
Melanconis marginalis subsp. europaea	CBS 131692*	*Alnus incana*	MN784976	MN784976	MN780759	MN780788	MN780816	Jaklitsch and Voglmayr (2020)
Melanconis marginalis subsp. europaea	CBS 131694	*Alnus alnobetula*	MN784977	MN784977	MN780760	MN780789	MN780817	Jaklitsch and Voglmayr (2020)
Melanconis marginalis subsp. europaea	MAV1	*Alnus alnobetula*	MN784978	MN784978	MN780761	MN780790	MN780818	Jaklitsch and Voglmayr (2020)
Melanconis marginalis subsp. italica	MFLUCC 16-1199*	*Alnus cordata*	MF190151	MF190096	NA	NA	NA	Senanayake et al. (2017)
Melanconis marginalis subsp. italica	MFLUCC 17-1659*	*Alnus cordata*	MF190152	MF190097	MF377602	NA	NA	Senanayake et al. (2017)
Melanconis marginalis subsp. marginalis	D321*	Alnus alnobetula subsp. crispa	MN784979	MN784979	MN780762	MN780791	MN780819	Jaklitsch and Voglmayr (2020)
Melanconis marginalis subsp. marginalis	D321a*	Alnus alnobetula subsp. crispa	MN784980	MN784980	MN780763	MN780792	MN780820	Jaklitsch and Voglmayr (2020)
Melanconis marginalis subsp. marginalis	D321b*	Alnus alnobetula subsp. crispa	MN784981	MN784981	MN780764	MN780793	MN780821	Jaklitsch and Voglmayr (2020)
Melanconis marginalis subsp. marginalis	CBS 109496	Alnus alnobetula subsp. maximowiczii	MN784982	MN784982	MN780765	MN780794	MN780822	Jaklitsch and Voglmayr (2020)
Melanconis marginalis subsp. marginalis	AR 4864	*Alnus alnobetula*	MN784983	MN784983	MN780766	MN780795	MN780823	Jaklitsch and Voglmayr (2020)
Melanconis marginalis subsp. marginalis	CBS 133346	*Alnus alnobetula*	MN784984	MN784984	MN780767	MN780796	MN780824	Jaklitsch and Voglmayr (2020)
Melanconis marginalis subsp. marginalis	MAFF 410218	Alnus alnobetula subsp. maximowiczii	MN784985	MN784985	MN780768	MN780797	MN780825	Jaklitsch and Voglmayr (2020)
Melanconis marginalis subsp. tirolensis	CBS 122310*	*Alnus alnobetula*	MN784986	MN784986	MN780769	MN780798	MN780826	Jaklitsch and Voglmayr (2020)
Melanconis marginalis subsp. tirolensis	D322a	*Alnus alnobetula*	MN959458	MN959458	MN989415	MN989416	MN989417	Jaklitsch and Voglmayr (2020)
*Melanconis pacifica*	CBS 109744	*Alnus rubra*	EU199197	AF408373	DQ862022	DQ862038	EU219103	Mejía et al. (2008)
*Melanconis stilbostoma*	D143	*Betula pendula*	KY427156	KY427156	KY427206	KY427225	KY427241	Voglmayr et al. (2017)
*Melanconis stilbostoma*	D258	*Betula aetnensis*	MN784987	MN784987	MN780770	MN780799	MN780827	Jaklitsch and Voglmayr (2020)
*Melanconis stilbostoma*	CBS 109778*	*Betula pendula*	MN784988	MN784988	MN780771	MN780800	MN780828	Jaklitsch and Voglmayr (2020)
*Melanconis stilbostoma*	CBS 121894	*Betula pendula*	KY427156	KY427156	JQ926302	JQ926368	MN780830	Voglmayr et al. (2017)
*Melanconis stilbostoma*	CBS 133338	*Betula papyrifera*	MN784990	MN784990	MN780773	MN780802	MN780831	Jaklitsch and Voglmayr (2020)
*Melanconis stilbostoma*	CFCC 50475	*Betula platyphylla*	KT732956	KT732975	KT732988	KT733005	KT733026	Fan et al. (2016a)
*Melanconis stilbostoma*	CFCC 50476	*Betula platyphylla*	KT732957	KT732976	KT732989	KT733006	KT733027	Fan et al. (2016a)
*Melanconis stilbostoma*	CFCC 50477	*Betula platyphylla*	KT732958	KT732977	KT732990	KT733007	KT733028	Fan et al. (2016a)
*Melanconis stilbostoma*	CFCC 50478	*Betula platyphylla*	KT732959	KT732978	KT732991	KT733008	KT733029	Fan et al. (2016a)
*Melanconis stilbostoma*	CFCC 50479	*Betula platyphylla*	KT732960	KT732979	KT732992	KT733009	KT733030	Fan et al. (2016a)
*Melanconis stilbostoma*	CFCC 50480	*Betula platyphylla*	KT732961	KT732980	KT732993	KT733010	KT733031	Fan et al. (2016a)
*Melanconis stilbostoma*	CFCC 50481	*Betula platyphylla*	KT732962	KT732981	KT732994	KT733011	KT733032	Fan et al. (2016a)
*Melanconis stilbostoma*	CFCC 50482	*Betula platyphylla*	KT732963	KT732982	KT732995	KT733012	KT733033	Fan et al. (2016a)
*Melanconis stilbostoma*	CFCC 50483	*Betula platyphylla*	KT732964	KT732983	KT732996	KT733013	KT733034	Fan et al. (2016a)
***Melanconis stilbostoma***	**CFCC 71570**	***Betula pendula***	**PX227557**	**PX227513**	**NA**	**PX233623**	**PX233632**	**In this study**
***Melanconis stilbostoma***	**CFCC 71571**	***Betula pendula***	**PX227558**	**PX227514**	**NA**	**PX233624**	**PX233633**	**In this study**
***Melanconis stilbostoma***	**CFCC 71572**	***Betula pendula***	**PX227559**	**PX227515**	**NA**	**PX233625**	**PX233634**	**In this study**
***Melanconis stilbostoma***	**CFCC 71573**	***Betula pendula***	**PX227560**	**PX227516**	**NA**	**PX233626**	**PX233635**	**In this study**
***Melanconis stilbostoma***	**CFCC 71574**	***Betula pendula***	**PX227561**	**PX227517**	**NA**	**PX233627**	**PX233636**	**In this study**

**Note.** “NA” indicates unavailable sequences, sequences produced in the current study are in bold and * means ex-type strains.

Phylogenetic reconstructions were performed on concatenated sequence datasets using both Maximum Likelihood (ML) and Bayesian Inference (BI) approaches. The datasets consisted of: (1) ITS-LSU-*rpb2*-*tef1* for *Coryneum*, (2) ITS-*act*-*rpb2*-*tef1-tub2* for *Cytospora*, (3) ITS-*tef1*-*tub2* for *Cryptosporella* and (4) ITS-LSU-*rpb2*-*tef1-tub2* for *Melanconis*. For the ML analysis, we employed the GTRGAMMA substitution model and conducted 1000 bootstrap replicates using RAxML-HPC v.8, implemented through the CIPRES Science Gateway portal (https://www.phylo.org/). The Bayesian analysis was performed using partition-specific evolutionary models selected with MrModelTest v.2.3, based on the Akaike Information Criterion (AIC). Markov Chain Monte Carlo (MCMC) simulations were run in MrBayes v.3.1.2 (Ronquist and Huelsenbeck 2003) with two independent runs of 10 million generations each, starting from random trees. We confirmed run convergence by monitoring the average standard deviation of split frequencies (< 0.01) and sampled trees every 1000 generations. After discarding the first 25% of trees as burn-in, posterior probabilities (PP) were calculated from the remaining trees. Nodal support was assessed using bootstrap support (BS) values (ML) from 1000 replicates and Bayesian posterior probabilities (PP). The resulting phylogenetic trees were visualised using FigTree v.1.4.4 (Rambaut 2018).

## ﻿Results

### ﻿Phylogenetic analyses

**Figure 1.**
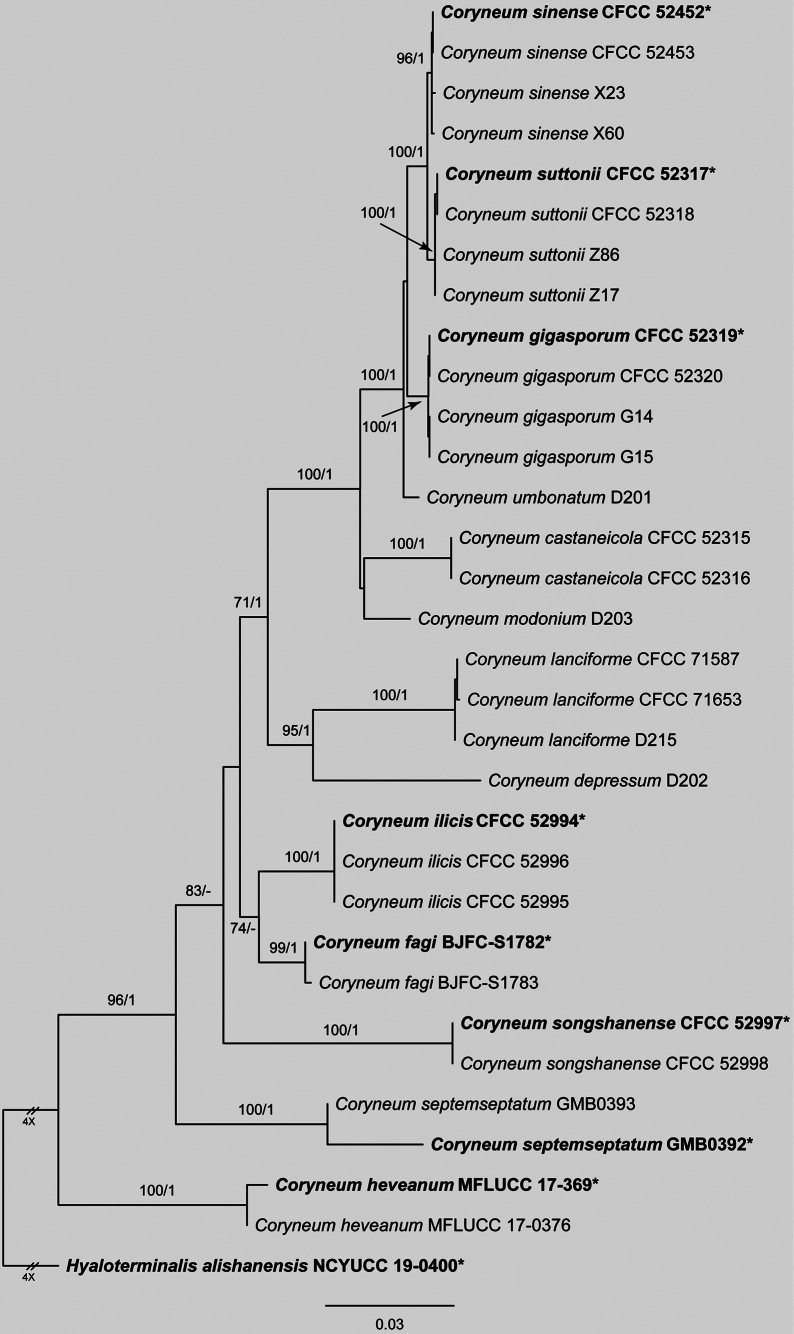
Maximum Likelihood tree of *Coryneum* generated from combined ITS, LSU, *rpb2* and *tef1* sequence data. Bootstrap support values ≥ 50% and Bayesian posterior probabilities ≥ 0.90 are demonstrated at the branches. Ex-type strains are marked in bold.

The concatenated alignment of ITS, LSU, *rpb2* and *tef1* for *Coryneum* included 32 strains and 3,380 characters (ITS: 1–630; LSU: 631–1,471; *rpb2*: 1,472–2,550; *tef1*: 2,551–3,380), with gaps retained. ML analysis yielded an optimal tree (likelihood = -13,031.13), with the alignment exhibiting 633 distinct patterns and 44.19% undetermined characters or gaps. Nucleotide frequencies were A = 0.240, C = 0.266, G = 0.283, T = 0.211 and substitution rates were AC = 1.258, AG = 2.217, AT = 1.350, CG = 1.193, CT = 7.495, GT = 1.0 (α = 0.140). BI employed models TNe+G4 (ITS), TN+F+I (LSU), TN+F+G4 (*rpb2*) and TN+F+G4 (*tef1*), with results matching ML topology. Isolates CFCC 71587 and CFCC 71653 clustered robustly with strain D215, confirming their placement as *Coryneum lanciforme* (Fig. 1).

**Figure 2.**
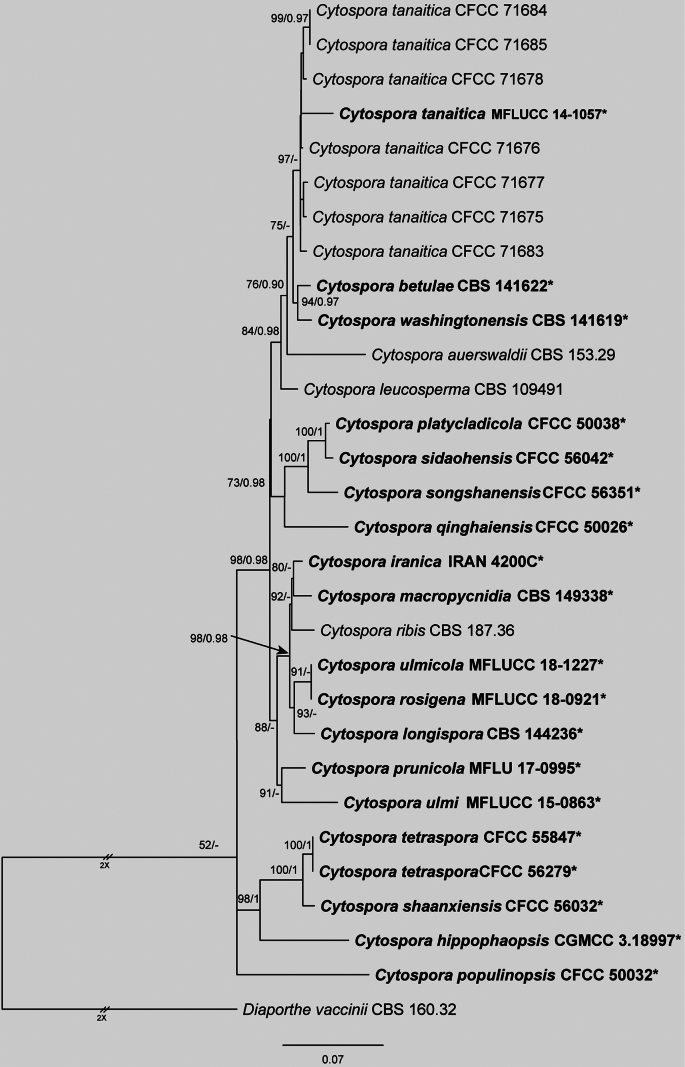
Maximum Likelihood tree of *Cytospora ribis* species complex generated from combined ITS, *act*, *rpb2*, *tef1* and *tub2* sequence data. Bootstrap support values ≥ 50% and Bayesian posterior probabilities ≥ 0.90 are demonstrated at the branches. Ex-type strains are marked in bold.

The concatenated dataset of ITS, *act*, *rpb2*, *tef1* and *tub2* for *Cytospora ribis* species complex, which contained CFCC 71675, CFCC 71676, CFCC 71677, CFCC 71678, CFCC 71683, CFCC 71684 and CFCC 71685, comprised 30 strains, spanning 2,818 aligned characters (*act*: 1–237; ITS: 238–742; *rpb2*: 743–1,940; *tef1*: 1,941–2,415; *tub2*: 2,416–2,818), including gaps. Maximum Likelihood (ML) analysis of this dataset produced a best-scoring tree with a likelihood value of −10563.20. The alignment matrix contained 711 distinct patterns, with 25.23% of sites representing undetermined characters or gaps. Nucleotide frequencies were estimated as follows: A = 0.248, C = 0.282, G = 0.236, T = 0.232. Substitution rates were AC = 1.417, AG = 3.402, AT = 1.463, CG = 0.735, CT = 6.621 and GT = 1.0, with a gamma distribution shape parameter (α) of 0.245. For Bayesian Inference (BI), the optimal evolutionary models, selected by MrModelTest, were TIM2e+G4 for ITS, K2P+G4 for *act*, TNe+R2 for *rpb2*, TIM2e+G4 for *tef1* and HKY+F+G4 for *tub2*. BI results were congruent with ML topology. Phylogenetic reconstruction placed the seven isolates from this study (CFCC 71675, CFCC 71676, CFCC 71677, CFCC 71678, CFCC 71683, CFCC 71684 and CFCC 71685) into a well-supported clade with MFLUCC 14-1057, identified as *Cytospora tanaitica* (Fig. 2).

**Figure 3.**
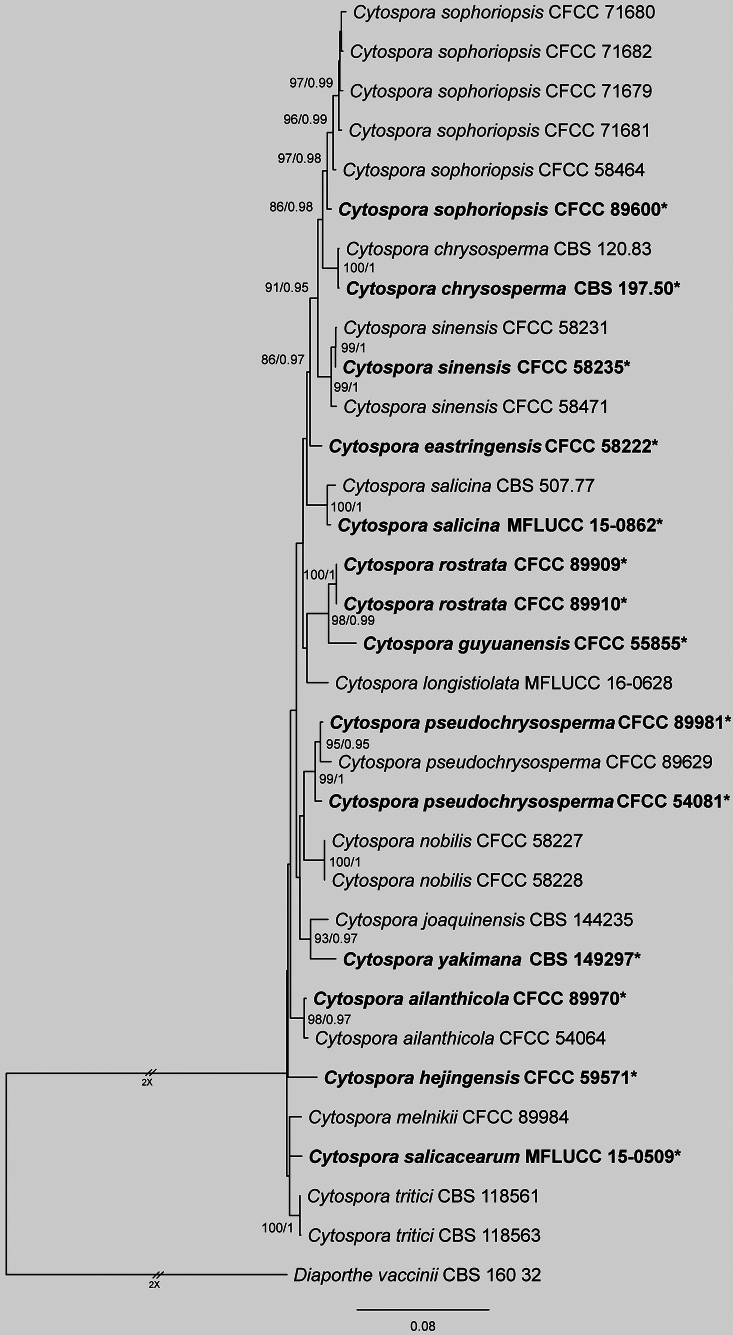
Maximum Likelihood tree of *Cytospora chrysosperma* species complex generated from combined ITS, *act*, *rpb2*, *tef1* and *tub2* sequence data. Bootstrap support values ≥ 50% and Bayesian posterior probabilities ≥ 0.90 are demonstrated at the branches. Ex-type strains are marked in bold.

The concatenated dataset of ITS, *act*, *rpb2*, *tef1* and *tub2* for *Cytospora chrysosperma* species complex, which contained CFCC 71679, CFCC 71680, CFCC 71681 and CFCC 71682, comprised 33 strains, spanning 3,182 aligned characters (*act*: 1–254; ITS: 255–754; *rpb2*: 755–1,952; *tef1*: 1,953–2,441; *tub2*: 2,442–3,182), including gaps. Maximum Likelihood (ML) analysis of this dataset produced a best-scoring tree with a likelihood value of -9050.97. The alignment matrix contained 608 distinct patterns, with 32.72% of sites representing undetermined characters or gaps. Nucleotide frequencies were estimated as follows: A = 0.248, C = 0.291, G = 0.236, T = 0.223. Substitution rates were AC = 1.133, AG = 3.787, AT = 1.739, CG = 0.964, CT = 7.756 and GT = 1.0, with a gamma distribution shape parameter (α) of 0.234. For Bayesian Inference (BI), the optimal evolutionary models, selected by MrModelTest, were TIM2e+G4 for ITS, K2P+G4 for *act*, TNe+R2 for *rpb2*, TIM2e+G4 for *tef1* and HKY+F+G4 for *tub2*. BI results were congruent with ML topology. Phylogenetic reconstruction placed the four isolates from this study (CFCC 71679, CFCC 71680, CFCC 71681 and CFCC 71682) into a well-supported clade with CFCC 58464 and CFCC 89600, identified as *Cytospora sophoriopsis* (Fig. 3).

**Figure 4.**
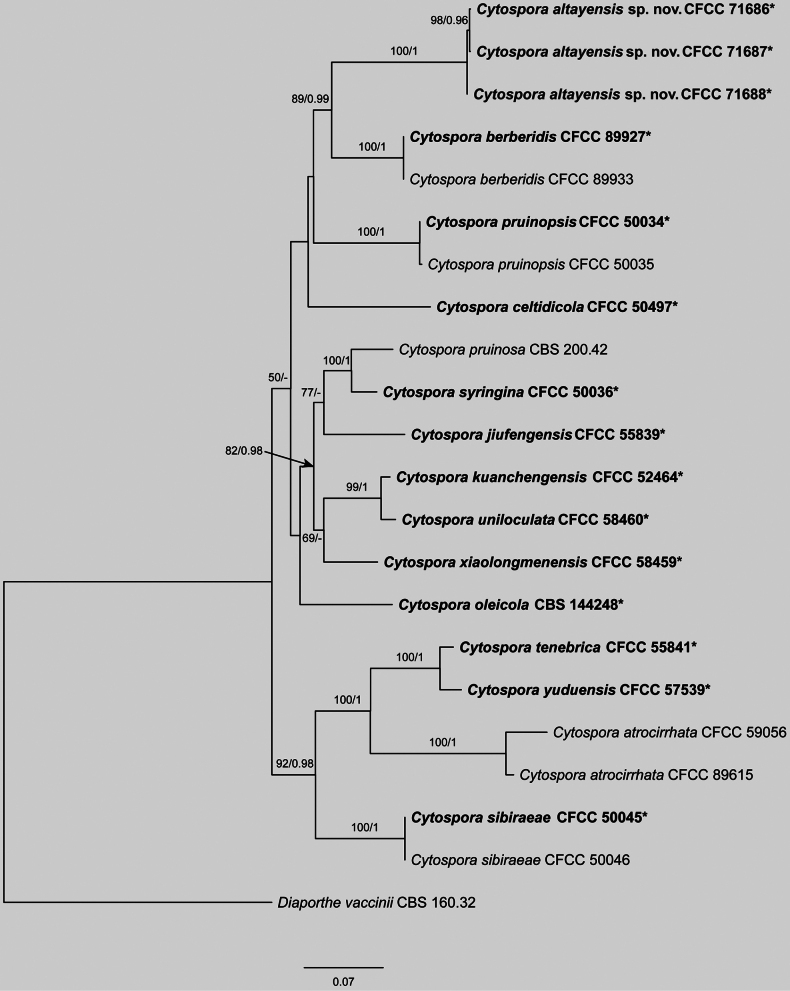
Maximum Likelihood tree of *Cytospora pruinosa* species complex generated from combined ITS, *act*, *rpb2*, *tef1* and *tub2* sequence data. Bootstrap support values ≥ 50% and Bayesian posterior probabilities ≥ 0.90 are demonstrated at the branches. Ex-type strains are marked in bold.

The concatenated dataset of ITS, *act*, *rpb2*, *tef1* and *tub2* for *Cytospora pruinosa* species complex, which contained CFCC 71686, CFCC 71687 and CFCC 71688, comprised 22 strains, spanning 3,198 aligned characters (*act*: 1–231; ITS: 232–815; *rpb2*: 816–2,006; *tef1*: 2,007–2,563; *tub2*: 2,564–3,198), including gaps. Maximum Likelihood (ML) analysis of this dataset produced a best-scoring tree with a likelihood value of -15361.07. The alignment matrix contained 1,056 distinct patterns, with 29.73% of sites representing undetermined characters or gaps. Nucleotide frequencies were estimated as follows: A = 0.242, C = 0.289, G = 0.241, T = 0.226. Substitution rates were AC = 2.020, AG = 4.309, AT = 2.146, CG = 1.435, CT = 8.769 and GT = 1.0, with a gamma distribution shape parameter (α) of 0.273. For Bayesian Inference (BI), the optimal evolutionary models, selected by MrModelTest, were TNe+G4 for ITS, TNe+G4 for *act*, TNe+G4 for *rpb2*, TIM2+F+I+G4 for *tef1* and HKY+F+G4 for *tub2*. BI results were congruent with ML topology. Phylogenetic reconstruction placed the three isolates from this study (CFCC 71686, CFCC 71687 and CFCC 71688) into a well-supported clade distinct from the any other known species, identified as *Cytospora altayensis* sp. nov. (Fig. 4).

**Figure 5.**
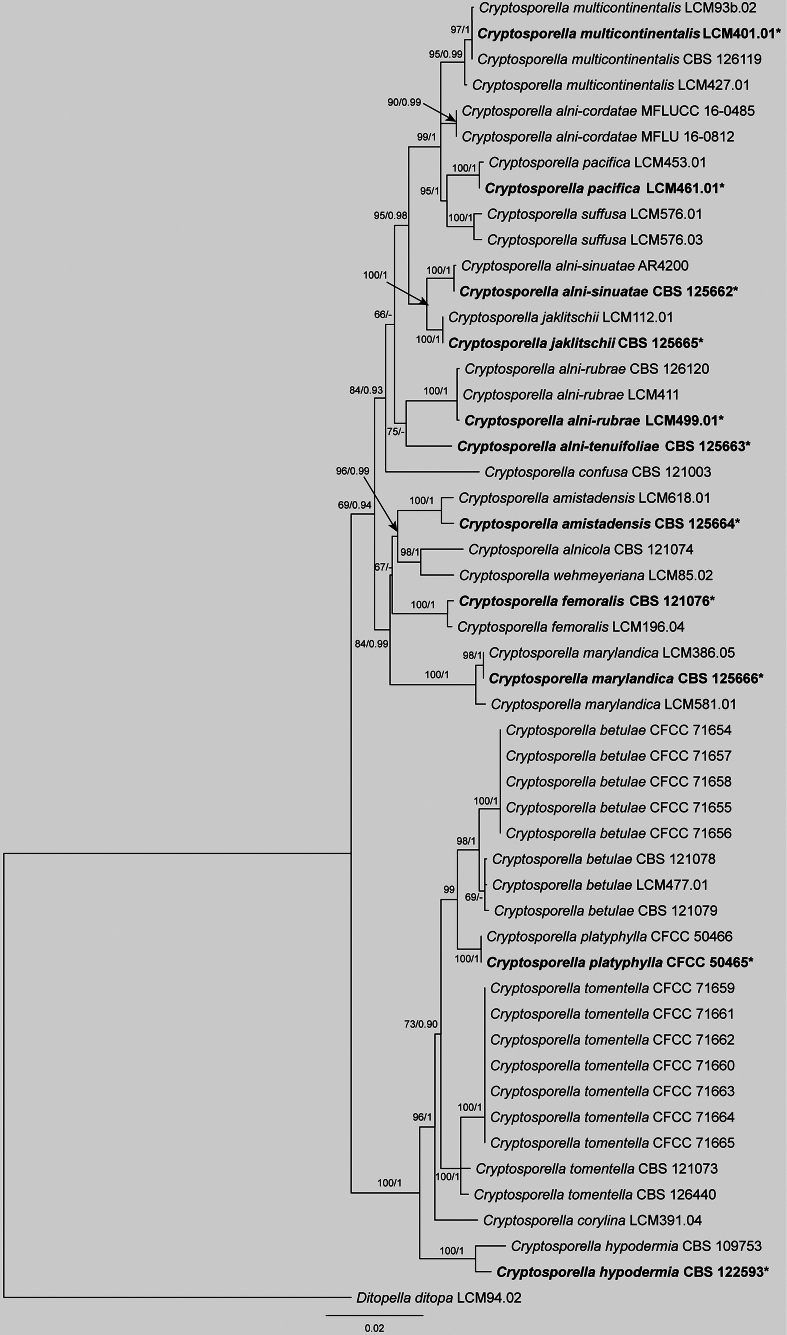
Maximum Likelihood tree of *Cryptosporella* generated from combined ITS, *tef1* and *tub2* sequence data. Bootstrap support values ≥ 50% and Bayesian posterior probabilities ≥ 0.90 are demonstrated at the branches. Ex-type strains are marked in bold.

The concatenated alignment of ITS, *tef1* and *tub2* for *Cryptosporella* included 51 strains and 3,130 characters (ITS: 1–576; *tef1*: 577–1,587; *tub2*: 1,588–3,130), with gaps retained. ML analysis yielded an optimal tree (likelihood = -9588.02), with the alignment exhibiting 664 distinct patterns and 30.57% undetermined characters or gaps. Nucleotide frequencies were A = 0.215, C = 0.306, G = 0.243, T = 0.234 and substitution rates were AC = 1.198, AG = 3.228, AT = 0.866, CG = 1.028, CT = 5.583, GT = 1.0 (α = 0.265). BI employed models K2P+R2 (ITS), TN+F+G4 (*tef1*), and HKY+F+G4 (*tub2*), with results matching ML topology. Twelve Isolates from this study clustered robustly into two clades with high support values, viz. CFCC 71654–71658, identified as *Cr. betulae* and CFCC 71659–71665, identified as *Cr. tomentella* (Fig. 5).

**Figure 6.**
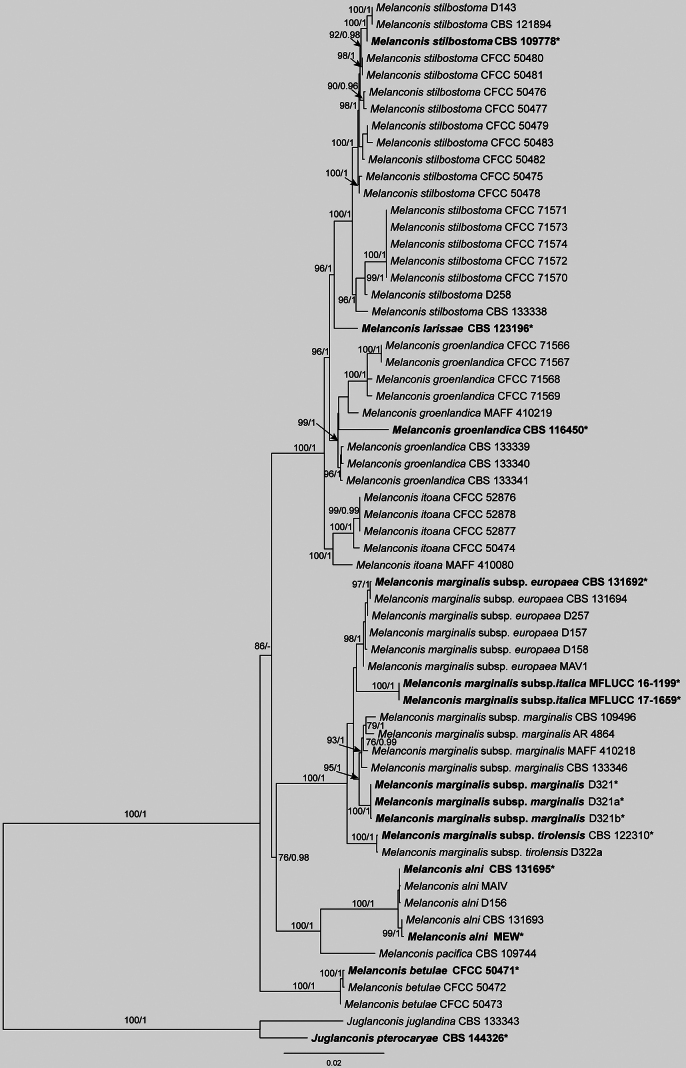
Maximum Likelihood tree of *Melanconis* generated from combined ITS, LSU, *rpb2*, *tef1* and *tub2* sequence data. Bootstrap support values ≥ 50% and Bayesian posterior probabilities ≥ 0.90 are demonstrated at the branches. Ex-type strains are marked in bold.

The concatenated alignment of ITS, LSU, *rpb2*, *tef1* and *tub2* for *Melanconis* included 62 strains and 5,659 characters (ITS: 1–725; LSU: 726–1,568; *rpb2*: 1,569–2,727; *tef1*: 2,728–4,083; *tub2*: 4,084–5,659), with gaps retained. ML analysis yielded an optimal tree (likelihood = -17771.87), with the alignment exhibiting 968 distinct patterns and 29.12% undetermined characters or gaps. Nucleotide frequencies were A = 0.233, C = 0.275, G = 0.265, T = 0.225 and substitution rates were AC = 1.580, AG = 3.397, AT = 1.699, CG = 1.061, CT = 8.067, GT = 1.0 (α = 0.140). BI employed models TIM2e+R2 (ITS), TIM2e+I (LSU), TNe+G4 (*rpb2*), TIM2+F+G4 (*tef1*) and HKY+F+G4 (*tub2*), with results matching ML topology. Nine Isolates from this study clustered robustly into two clades with high support values, viz. CFCC 71566–71569, identified as *M. groenlandica* and CFCC 71570–71574, identified as *M. stilbostoma* (Fig. 6).

### ﻿Taxonomy

#### ﻿ Coryneaceae

Taxon classificationFungiDiaporthales﻿Coryneaceae

Corda, Icon. fung. (Prague) 3: 36 (1839)

F8AD843B-C13B-579F-A5F5-B3DDDC7CCDAF

##### Notes.

Coryneaceae, typified by *Coryneum*, represents a both morphologically and phylogenetically distinct lineage within Diaporthales, primarily characterised by the production of transversely distoseptate, brown conidia (Senanayake et al. 2017, 2018; Jiang et al. 2018, 2019; Rathnayaka et al. 2020). To date, three genera have been accepted in Coryneaceae, based on integrated morphological and phylogenetic evidence, viz. *Coryneum*, *Hyaloterminalis* and *Subellipsoidispora* (Tang et al. 2023). While phylogenetic analyses have placed *Talekpea* in close relationship with *Coryneum* (Rathnayaka et al. 2020; Tang et al. 2023), this genus exhibits significant morphological divergence from the three accepted genera. Resolution of its taxonomic status will require additional sampling and more comprehensive studies in the future. Notably, *Hyaloterminalis* is distinguished from *Coryneum*, based on its pycnidial conidiomata (Rathnayaka et al. 2020). *Subellipsoidispora* has been described only from its sexual morph, distinguished from *Coryneum* by ascomata typically bearing a single peridium (Tang et al. 2023). This disparity in known morphologies highlights the need for further research to fully understand the life histories and evolutionary relationships within Coryneaceae.

#### ﻿ Coryneum

Taxon classificationFungiDiaporthales﻿Coryneaceae

Nees, Syst. Pilze (Würzburg): 34 (1816)

6C01FA7E-1F95-5911-8157-FFADAB1AF687

= Pseudovalsa Ces. & De Not., Comm. Soc. crittog. Ital. 1(fasc. 4): 206 (1863).

##### Notes.

*Coryneum*, typified by *C. umbonatum*, is the largest genus within the family Coryneaceae. The asexual morphs of *Coryneum* are commonly observed and are predominantly found on tree genera such as *Betula*, *Castanea* and *Quercus* (Senanayake et al. 2017; Jiang et al. 2018, 2019). Species in this genus are characterised by transversely distoseptate, brown conidia (Senanayake et al. 2017). Currently, the delimitation of species within *Coryneum* relies on a combination of morphological characteristics and molecular phylogenetic analyses, based on ITS, LSU, *rpb2* and *tef1* loci (Jiang et al. 2018, 2019). Nevertheless, the lack of molecular data for many described species remains a constraint for accurate species identification (Jiang et al. 2018, 2019). Species of *Coryneum* are commonly reported as plant pathogen causing canker diseases and some species are saprobic on decaying wood contributing to nutrient cycling (Senanayake et al. 2017, 2018).

#### ﻿ Coryneum lanciforme

Taxon classificationFungiDiaporthales﻿Coryneaceae

(Fr.) Voglmayr & Jaklitsch, IMA Fungus 6(1): 146 (2015)

29E4A562-B55A-50F8-9EF4-4151CEA90FA4

Fig. 7

= Coryneum brachyurum Link, Sp. pl., Edn 4 6(2): 124 (1825).
≡ Pseudovalsa lanciformis (Fr.) Ces. & De Not., Comm. Soc. crittog. Ital. 1(fasc. 4): 206 (1863).

##### Description.

***Pseudostromata*** semi-immersed in the bark, scattered, conical, 950–1700 μm diam., 600–900 μm high, with 3–8 perithecia arranged irregularly. ***Ectostromatic*** disc distinct, circular, brown, 430–650 μm diam. ***Ostioles*** black, 75–140 μm diam. ***Perithecia*** globular, somewhat flattened at base with black neck, 350–650 μm diam. ***Asci*** hyaline, with chitinoid, refractive ring, clavate to elongate-obovoid, (190–)207–241(–246) × (30–)33–40(–42) μm, 8-spored, biseriate. ***Ascospores*** fusiform, ends pointed, dark brown, 5–8-distoseptate, (37.5–)39.5–48.5(–53) × (13.5–)14–17(–18.5) (av. = 44.1 ± 4.4 × 15.4 ± 1.6, n = 50) (n = 50) μm, L/W ratio = 2.4–3.6.

**Figure 7.**
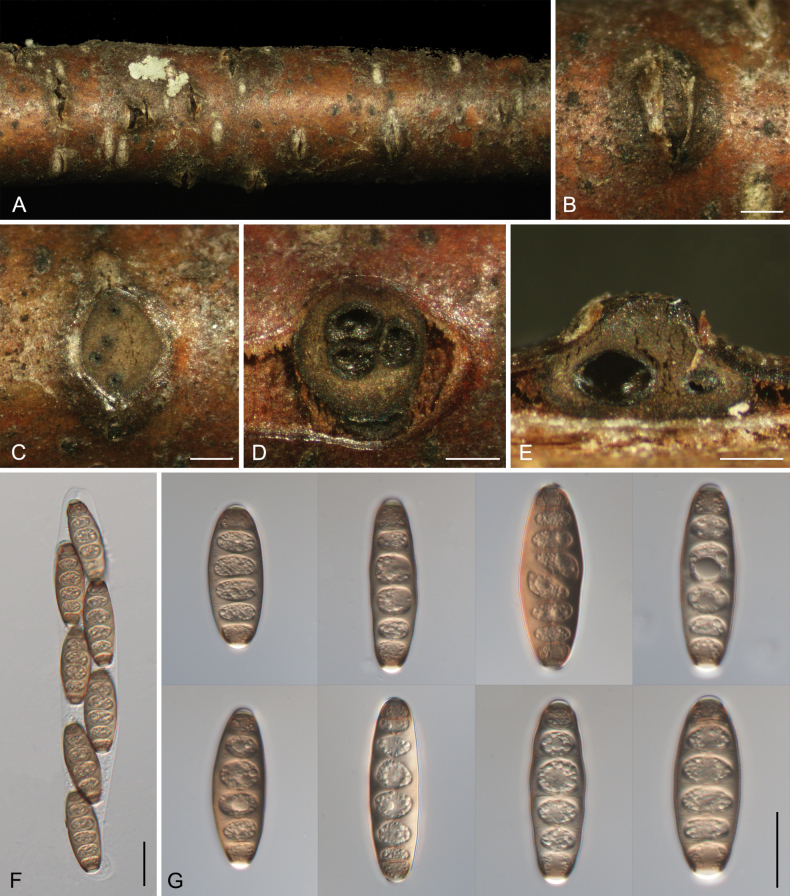
*Coryneum lanciforme* from *Betula pendula*. **A, B.** Habit of ascostromata on branch; **C.** Ostioles embedded in ascostroma in section; **D.** Transverse section through ascostroma; **E.** Longitudinal section through ascostroma; **F.** Asci; **G.** Ascospores. Scale bars: 500 μm (**B–E**); 20 μm (**F, G**).

##### Culture characteristics.

***Colonies*** on PDA flat, spreading, with moderate flocculent aerial mycelium and undulating margin, initially white, becoming dark brown and reaching 90 mm diam. after 3 weeks at 25 °C.

##### Materials examined.

**China** • Xinjiang Uygur Autonomous Region, Altay Prefecture, Altay City, Alahake Town, Haxionggou, from dead branches of *Betula pendula*, 7 October 2024, Rong Ma, Caixia Wang & Hailong Lu (XJAU 4053, living cultures CFCC 71587 and CFCC 71653).

##### Notes.

*Coryneum lanciforme* is the type species of *Pseudovalsa* (Sutton 1975). However, since *Coryneum umbonatum* (the type species of *Coryneum*) was described earlier (Nees von Esenbeck 1816), *Pseudovalsa* was synonymised under *Coryneum* and *P. lanciformis* was treated as a synonym of *Co. lanciforme* (Rossman et al. 2015). In this study, we report the first discovery of *Co. lanciforme* along with its teleomorph in Xinjiang, China, significantly expanding the known geographical distribution of this fungus.

#### ﻿ Cytosporaceae

Taxon classificationFungiDiaporthalesCytosporaceae

Fr. [as ‘Cytisporei’], Syst. orb. veg. (Lundae): 118 (1825)

88DA98D1-DC98-5F39-A5C8-DF64A0A02E9A

##### Notes.

Cytosporaceae is a morphological and phylogenetical family of Diaporthales, containing a single genus *Cytospora* (Lin et al. 2024).

#### ﻿ Cytospora

Taxon classificationFungiDiaporthalesCytosporaceae

Ehrenb., Sylv. mycol. berol. (Berlin): 28 (1818)

8CC67AEF-D989-5597-BDD6-B0466CE3911C

##### Notes.

*Cytospora* is characterised by the single or labyrinthine, loculate stromata, filamentous conidiophores and allantoid hyaline conidia and ascospores (Jiang et al. 2020b; Zhu et al. 2020; Cai et al. 2024; Jia et al. 2024; Li et al. 2024; Lin et al. 2024; Jiang et al. 2025a; Ilyukhin et al. 2025). Members of this genus are commonly known as causal agents of tree canker diseases, but also include endophytic and saprophytic species (Fan et al. 2020).

#### ﻿ Cytospora altayensis

Taxon classificationFungiDiaporthalesCytosporaceae

C.X. Wang, Ning Jiang & R. Ma sp. nov.

1BD15686-3BA8-5FD2-88CB-087023568051

860537

Fig. 8

##### Etymology.

Named after the collection site of the holotype, Altay Prefecture.

##### Description.

***Conidiomata*** pycnidial, scattered, immersed to semi-immersed in the bark, discoid to conical, 400–600 μm diam., 250–400 μm high, with an undivided locule. ***Conceptacle*** absent. ***Ectostromatic*** disc isabelline, circular to ovoid, 150–250 μm diam., with a single ostiole per disc in the centre. ***Ostiole*** grey to black, 50–100 μm diam. ***Conidiophores*** borne along the locules, hyaline, unbranched or branched at the base, (29–)35.5–43.5(–47) × 1.5–2 μm. ***Conidiogenous cells*** enteroblastic, phialidic, subcylindrical to cylindrical. ***Conidia*** hyaline, allantoid, thin-walled, eguttulate, aseptate, smooth, (8.5–)9.5–11.5(–13) × 2.5–3(–3.5) (av. = 10.6 ± 1 × 2.8 ± 0.2, n = 50) μm, L/W ratio = 3.4–4.2.

**Figure 8.**
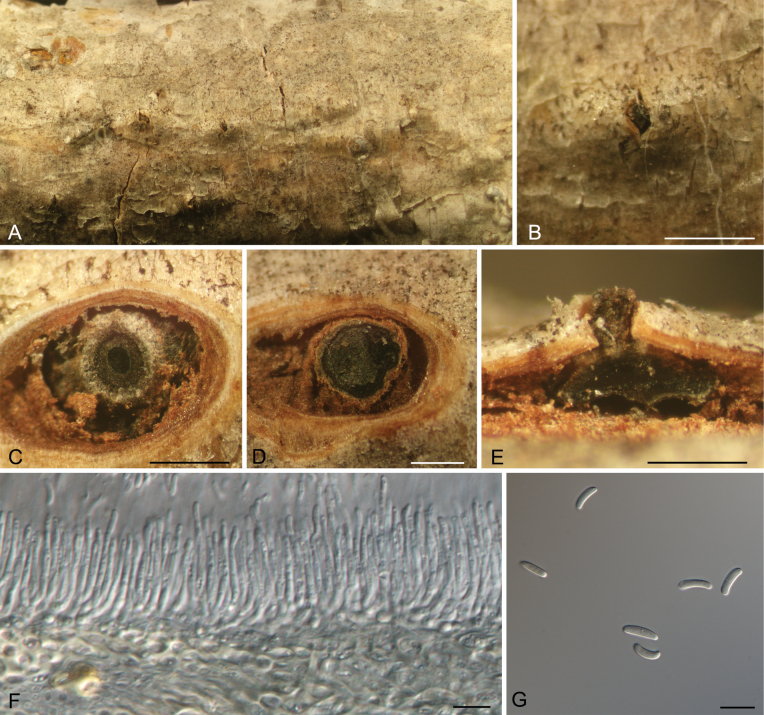
Morphology of *Cytospora altayensis* from *Betula pendula*. **A, B.** Habit of conidiomata on branch; **C, D.** Transverse section through conidiomata; **E.** Longitudinal section through conidiomata; **F.** Conidiophores and conidiogenous cells; **G.** Conidia. Scale bars: 300 μm (**B–E**); 10 μm (**F, G**).

##### Culture characteristics.

***Colonies*** on PDA flat, spreading, with moderate aerial mycelium and undulating margin, saffron to ochreous, reaching 90 mm diam. after 2 weeks at 25 °C, sterile.

##### Materials examined.

**China** • Xinjiang Uygur Autonomous Region, Altay Prefecture, Habahe County, Birch Forest Scenic Area, from branches of *Betula pendula*, 4 October 2024, Rong Ma, Caixia Wang & Hailong Lu (holotype XJAU 3988, ex-holotype cultures CFCC 71686, CFCC 71687 and CFCC 71688).

##### Notes.

Three isolates of *Cytospora* from *Betula pendula* in this study formed a distinct subclade phylogenetically close to *Cy. berberidis* from *Berberis dasystachya*, representing a new species (Fig. 4). However, *Cy. altayensis* can be distinguished from *Cy. berberidis* by conidial size (9.5–11.5 × 2.5–3 μm in *Cy. berberidis* vs. 6–6.9 × 1.9–2.1 μm in *Cy. berberidis*) (Liu et al. 2015). At the nucleotide level, *Cy. altayensis* differs from *Cy. berberidis* (ITS, 15/581; *act*, 22/254; *rpb2*, 58/721; *tef1*, 161/597; *tub2*, 75/415) (Jeewon and Hyde 2016).

#### ﻿ Cytospora sophoriopsis

Taxon classificationFungiDiaporthalesCytosporaceae

X.L. Fan & C.M. Tian, Persoonia 45: 39 (2019).

E961D39C-A5AB-5747-837D-D36724EF0440

##### Description.

See Fan et al. (2020).

##### Materials examined.

**China** • Xinjiang Uygur Autonomous Region, Altay Prefecture, Altay City, Alahake Town, Haxionggou, from branches of *Betula pendula*, 7 October 2024, Rong Ma, Caixia Wang & Hailong Lu (XJAU 4048, cultures CFCC 71679, CFCC 71680, CFCC 71681 and CFCC 71682).

##### Notes.

*Cytospora sophoriopsis* was introduced on cankered branches of *Styphnolobium japonicum* (Fan et al. 2020). This fungus was later confirmed as poplar and willow canker pathogens in China (Lin et al. 2022, 2023). In this study, we firstly discovered this species from *Betula pendula* in XUAR (Fig. 3).

#### ﻿ Cytospora tanaitica

Taxon classificationFungiDiaporthalesCytosporaceae

Norph., Bulgakov & K.D. Hyde, Fungal Diversity 75: 172 (2015)

87199035-F4C0-5EEA-A7DA-9C4EE1F1BA06

Fig. 9

##### Description.

***Conidiomata*** pycnidial, serried, semi-immersed in the bark, conical, 1200–2000 μm diam., 400–650 μm high, with multiple subdivided locules with common walls. ***Conceptacle*** absent. ***Ectostromatic disc*** honey, circular to ovoid, 350–650 μm diam., with a single ostiole per disc in the centre. ***Ostiole*** grey to black, 75–140 μm diam. ***Conidiophores*** borne along the locules, hyaline, branched, (13–)15–28.5(–32) × 1.5–2 μm. ***Conidiogenous cells*** enteroblastic, phialidic, subcylindrical to cylindrical. ***Conidia*** hyaline, allantoid, thin-walled, eguttulate, aseptate, smooth, (5–)5.5–6(–7) × 1.5–2 (av. = 5.8 ± 0.4 × 1.7 ± 0.1, n = 50) μm, L/W ratio = 3.2–3.8.

##### Culture characteristics.

***Colonies*** on PDA flat, spreading, with abundant aerial mycelium and rough margin, grey to dark green, reaching 90 mm diam. after 2 weeks at 25 °C, sterile.

##### Materials examined.

**China** • Xinjiang Uygur Autonomous Region, Altay Prefecture, Altay City, Alahake Town, Haxionggou, from branches of *Betula pendula*, 7 October 2024, Rong Ma, Caixia Wang & Hailong Lu (XJAU 4047, cultures CFCC 71675, CFCC 71676); • Xinjiang Uygur Autonomous Region, Altay Prefecture, Altay City, from branches of *Betula pendula*, 7 October 2024, Rong Ma, Caixia Wang & Hailong Lu (XJAU 4064, cultures CFCC 71677 and CFCC 71678); • Xinjiang Uygur Autonomous Region, Altay Prefecture, Qinghe County, Xileasheke Village, from branches of *Betula pendula*, 8 October 2024, Rong Ma, Caixia Wang & Hailong Lu (XJAU 4084, cultures CFCC 71683, CFCC 71684, CFCC 71685).

**Figure 9.**
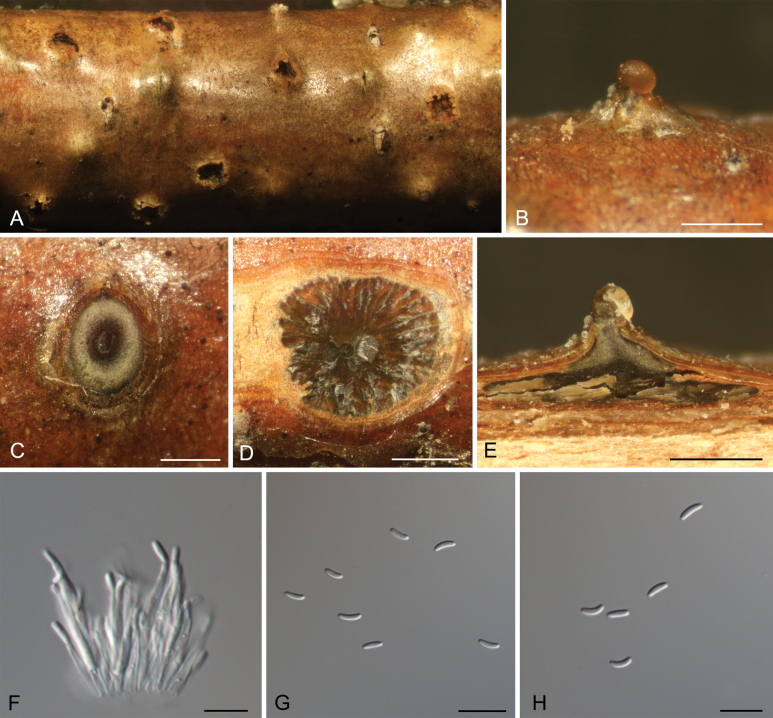
Morphology of *Cytospora tanaitica* from *Betula pendula*. **A, B.** Habit of conidiomata on branch; **C, D.** Transverse section through conidiomata; **E.** Longitudinal section through conidiomata; **F.** Conidiophores and conidiogenous cells; **G.** Conidia. Scale bars: 500 μm (**B–E**); 10 μm (**F–G**).

##### Notes.

*Cytospora tanaitica* was first described from Betula pubescens var. glabrata in Russia (Ariyawansa et al. 2015). In the present study, seven isolates obtained from *Betula pendula* in Xinjiang, China, clustered within a single clade together with the ex-type strain of *Cy. tanaitica* in the phylogenetic tree (Fig. 2). However, the conidia of our specimens were larger than those of the holotype (5.5–6 × 1.5–2 μm vs. 3.5–4 × 0.6–0.7 μm) (Ariyawansa et al. 2015). Based on the molecular phylogenetic evidence and the shared host genus, the isolates obtained in this study are identified as *Cy. tanaitica*, representing a new host and geographic record for this species.

#### ﻿ Gnomoniaceae

Taxon classificationFungiDiaporthales﻿Gnomoniaceae

G. Winter [as ‘Gnomonieae’], Rabenh. Krypt.-Fl. 1(2): 570 (1886)

54CA5752-7259-5C69-8EDD-7EC7424F23A8

##### Notes.

Gnomoniaceae is characterised by immersed, rarely erumpent or superficial ascomata, without a stroma or aggregated with a rudimentary stroma (Senanayake et al. 2017). Members of Gnomoniaceae are endophytes, pathogens and saprobes inhabiting various hosts and substrates (Senanayake et al. 2018).

#### ﻿ Cryptosporella

Taxon classificationFungiDiaporthales﻿Gnomoniaceae

Sacc., Michelia 1(no. 1): 30 (1877)

22FF7E61-D698-5C95-818A-07D1AC3F91D4

##### Notes.

*Cryptosporella* is characterised by aggregated ascomata below the bark surface, with converging necks and ellipsoid to elongated, aseptate or rarely 1-septate ascospores (Mejía et al. 2008, 2011). Species of this genus are usually distributed in temperate regions as endophytes and occasionally as saprobes and pathogens on hardwood trees, such as Betulaceae, Tiliaceae and Ulmaceae (Barr 1978; Mejía et al. 2011; Fan et al. 2016 b).

#### ﻿ Cryptosporella betulae

Taxon classificationFungiDiaporthales﻿Gnomoniaceae

(Tul. & C. Tul.) L.C. Mejía & Castl., Mycol. Res. 112(1): 32 (2008)

D28CB89E-A953-556D-B23D-D03FF0E3FDDD

Fig. 10

##### Description.

***Conidiomata*** acervular, immersed to semi-immersed in the bark, scattered, conical, 850–1200 μm diam., 650–800 μm high. ***Central column*** beneath the disc more or less conical, grey to black. ***Ectostromatic disc*** brown, circular to ovoid, 350–420 μm diam. ***Conidiophores*** reduced to conidiogenous cells. ***Conidiogenous cells*** narrowly cylindrical, smooth, hyaline, producing a conidium at apex, (6–)8–10.5(–13) × (3–)4.5–6.5(–8) μm. ***Conidia*** hyaline, aseptate, cylindrical to clavate, curved, (52–)55.5–66.5(–70) × (4.5–)5.5–6.5 μm (av. = 61 ± 5.2 × 5.8 ± 0.6 μm, n = 50) μm, L/W ratio = 9.1–11.9.

##### Culture characteristics.

***Colonies*** on PDA flat, spreading, with moderate aerial mycelium and even margin, white to fawn, reaching 70 mm diam. after 2 weeks at 25 °C, sterile.

##### Materials examined.

**China** • Xinjiang Uygur Autonomous Region, Altay Prefecture, Altay City, Alahake Town, Haxionggou Valley, from branches of *Betula pendula*, 7 October 2024, Rong Ma, Caixia Wang & Hailong Lu (XJAU 4059 cultures CFCC 71654 and CFCC 71655); *ibid*. (XJAU 4060, cultures CFCC 71656, CFCC 71657 and CFCC 71658).

##### Notes.

The species concept of *Cryptosporella betulae* was conceived more narrowly than previous studies, with only the sexual morph described (Mejía et al. 2008, 2011). This fungus has been recorded in Austria and Russia inhabiting *Betula lenta* and *B. pendula* (Mejía et al. 2008, 2011). In this study, two new specimens collected from Xinjiang, China and five isolates were obtained. They were identified as *Cr. Betulae*, based on the molecular phylogeny (Fig. 5), representing a new host record in China. In addition, asexual morph of this fungus is firstly discovered and described herein.

**Figure 10.**
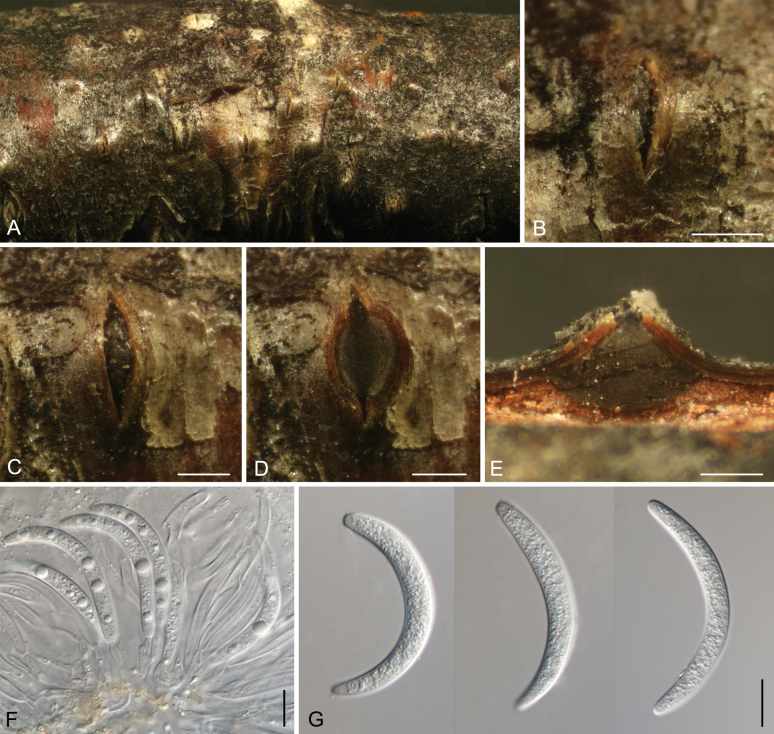
Morphology of *Cryptosporella betulae* from *Betula pendula*. **A–C.** Habit of conidiomata on branch; **D.** Transverse section through conidiomata; **E.** Longitudinal section through conidiomata; **F.** Conidiophores and conidiogenous cells; **G.** Conidia. Scale bars: 500 μm (**B–E**); 10 μm (**F, G**).

#### ﻿ Cryptosporella tomentella

Taxon classificationFungiDiaporthales﻿Gnomoniaceae

(Peck) L.C. Mejía, Mycologia 103(2): 397 (2011)

F95D8FD2-DE5D-509F-90BB-AD2AA64D90E4

Fig. 11

##### Description.

***Pseudostromata*** immersed to semi-immersed in the bark, scattered, conical, 1450–1800 μm diam., 450–600 μm high, with 8–15 perithecia arranged circularly or irregularly. ***Ectostromatic disc*** brown, circular to ovoid, 350–450 μm diam. ***Ostioles*** brown to black, 75–130 μm diam. ***Perithecia*** flask-shaped to spherical, 250–400 μm diam. ***Asci*** hyaline, without refractive ring, clavate, (85.5–)108–115(–126.5) × (9–)12.5–20(–21.5) μm, 8-spored. ***Ascospores*** 2–4-seriate, cylindrical, slightly curved, tapering towards rounded ends, thin-walled, hyaline, aseptate, (39–)47–61.5(–67) × (4.5–)5–5.5(–6.5) (av. = 54.2 ± 7 × 5.2 ± 0.6, n = 50) μm, L/W ratio = 9–11.8.

##### Culture characteristics.

***Colonies*** on PDA flat, spreading, with moderate aerial mycelium and undulate margin, yellowish, reaching 70 mm diam. after 2 weeks at 25 °C, sterile.

##### Materials examined.

**China** • Xinjiang Uygur Autonomous Region, Altay Prefecture, Jeminay County, Kizilkayin Red Birch Forest, from branches of *Betula microphylla*, 5 October 2024, Rong Ma, Caixia Wang & Hailong Lu (XJAU 4005, culture CFCC 71659); *ibid*. (XJAU 4006, cultures CFCC 71660, CFCC 71661 and CFCC 71662); *ibid*. (XJAU 4010, cultures CFCC 71663, CFCC 71664, CFCC 71665).

**Figure 11.**
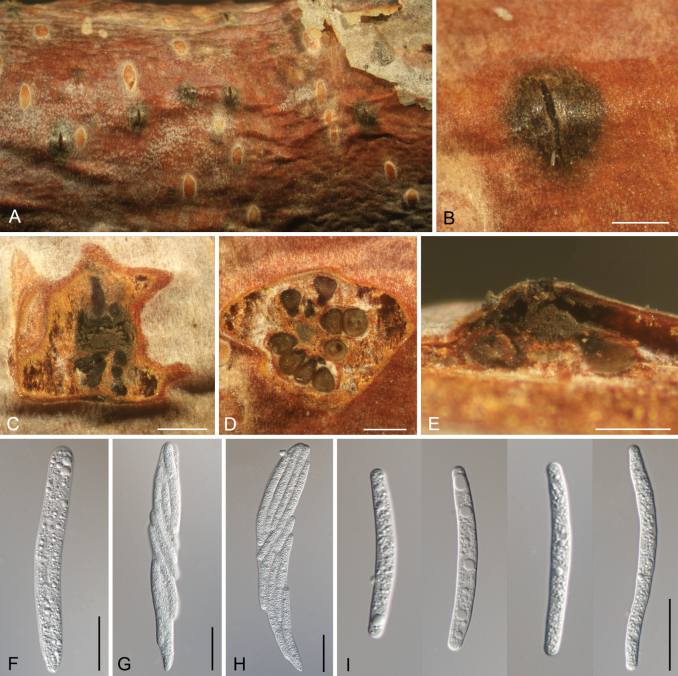
Morphology of *Cryptosporella tomentella* from *Betula microphylla*. **A, B.** Habit of ascostromata on branch; **C.** Ostioles embedded in ascostroma in section; **D.** Transverse section through ascostroma; **E.** Longitudinal section through ascostroma; **F–H.** Asci; **I.** Ascospores. Scale bars: 500 μm (**B–E**); 20 μm (**F–I**).

##### Notes.

*Cryptosporella tomentella* was previously regarded as a synonym of *Cr. betulae* (Reid and Booth 1987; Mejía et al. 2008). This species has been currently only documented in the United States, where it infects *Betula populifolia* and other *Betula* species (Mejía et al. 2008, 2011). The ascospore morphology of our specimens was consistent with that described for the *Cr. tomentella* lectotype (Mejía et al. 2011). In the present study, we report for the first time the occurrence of *Cr. tomentella* on *Betula microphylla* in China, supported by molecular phylogenetic evidence (Fig. 5).

#### ﻿ Melanconidaceae

Taxon classificationFungiDiaporthales﻿Melanconidaceae

G. Winter [as ‘Melanconideae’], Rabenh. Krypt.-Fl., Edn 2 (Leipzig) 1.2: 764 (1886)

A2CDA576-E712-5BFE-BFDF-3EAF4932D326

##### Notes.

Melanconidaceae was established by Winter (1886). This family initially encompassed numerous genera characterised by perithecia immersed in well-developed stromata, with ostioles emerging through an ectostromatic disc (Barr 1978). However, based on analyses of LSU sequence data, Castlebury et al. (2002) and Rossman et al. (2007) later reduced this family to include only the type genus, *Melanconis*. Fan et al. (2018b) further confirmed that Melanconidaceae should be treated as a monotypic family, containing only the genus *Melanconis*.

#### ﻿ Melanconis

Taxon classificationFungiDiaporthales﻿Melanconidaceae

Tul. & C. Tul., Select. fung. carpol. (Paris) 2: 115 (1863)

7A23E74B-94E7-5475-AD3B-E4E05D2CA254

##### Notes.

*Melanconis* is characterised by circularly arranged perithecia immersed in well-developed to reduced entostromata with a concolourous central column and ostioles erumpent through a light-coloured ectostromatic disc with hyaline, one-septate ascospores; acervuli with light-coloured central column producing brown, fusiform to pyriform alpha conidia and hyaline, cylindrical or allantoid beta conidia (Voglmayr et al. 2012; Fan et al. 2016a). All known species of this genus have been reported exclusively on hosts within the family Betulaceae (specifically *Alnus* and *Betula*) (Fan et al. 2018b; Jaklitsch and Voglmayr 2020).

#### ﻿ Melanconis groenlandica

Taxon classificationFungiDiaporthales﻿Melanconidaceae

(M. Bohn) L. Lombard & Crous, Persoonia 36: 234 (2016)

C9C91DB8-7520-5801-9E10-62B26F8694FF

Fig. 12

##### Description.

***Conidiomata*** acervular, semi-immersed in the bark, scattered, conical, 1050–1400 μm diam., 450–700 μm high. ***Central column*** beneath the disc conical, pale yellow. ***Ectostromatic disc*** dark brown to black, circular to ovoid, 400–450 μm diam. ***Conidiophores*** reduced to conidiogenous cells. ***Conidiogenous cells*** cylindrical, smooth, hyaline, producing a conidium at apex, (25–)32–38.5(–40) × 2.5–3.5(–4) μm. ***Alpha conidia*** brown, aseptate, ovoid, (8.5–)9.5–11(–11.5) × (6–)6.5–7.5(–8) (av. = 10.2 ± 0.7 × 7.1 ± 0.4, n = 50) μm, L/W ratio = 1.3–1.6.

##### Culture characteristics.

***Colonies*** on PDA flat, spreading, with sparse to moderate aerial mycelium and undulate margin, rosy to fawn, reaching 80 mm diam. after 2 weeks at 25 °C.

##### Materials examined.

**China** • Xinjiang Uygur Autonomous Region, Ili Kazakh Autonomous Prefecture, Zhaosu County, Xiata Ancient Trail National Forest Park, from branches of *Betula tianschanica*, 6 September 2023, Rong Ma, Chuli Liu, Caixia Wang & Wanbin Shi (XJAU 3812, cultures CFCC 71566, CFCC 71567, CFCC 71568, CFCC 71569).

**Figure 12.**
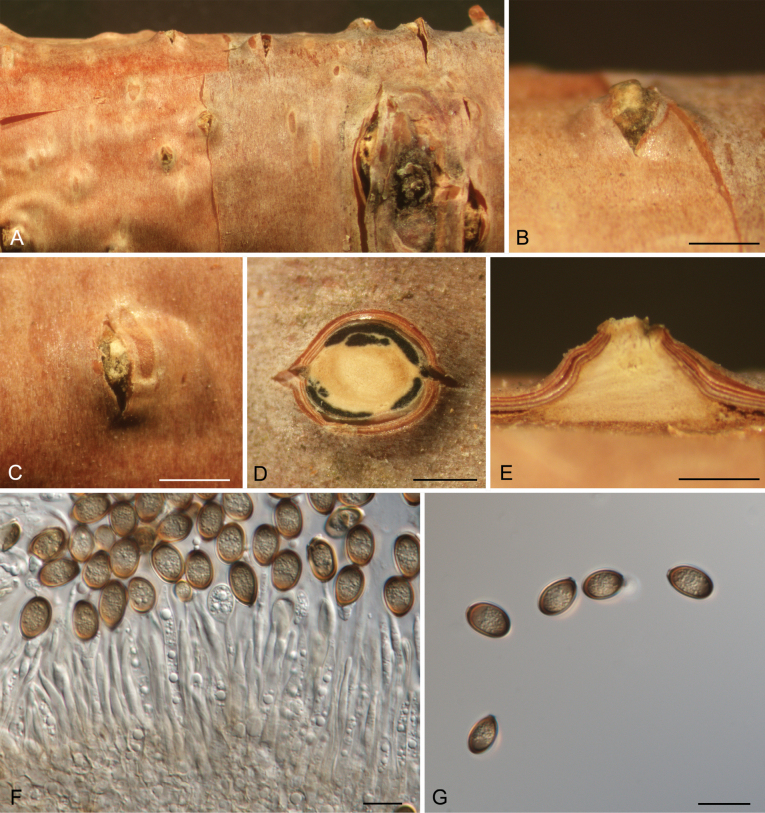
Morphology of *Melanconis groenlandica* from *Betula tianchanica*. **A–C.** Habit of conidiomata on branch; **D.** Transverse section through conidiomata; **E.** Longitudinal section through conidiomata; **F.** Conidiophores and conidiogenous cells; **G.** Alpha conidia. Scale bars: 500 μm (**B–E**); 10 μm (**F, G**).

##### Notes.

*Melanconis groenlandica* has been reported from the USA and Japan, where it occurs on *Betula maximowicziana*, *B. nana* and *B. papyrifera* (Bohn 1993; Jaklitsch and Voglmayr 2020). This fungus is known to exhibit an endophytic lifestyle in *Betula* hosts and its morphological characteristics were originally described in Bohn (1993) with illustrations (conidial size 9–15 × 5–7 μm). In the present study, we provide the first description of this species on its natural host, based on newly-collected specimens showing similar conidial characteristics to the holotype (Bohn 1993). Furthermore, this represents the first record of *M. groenlandica* on *Betula tianschanica* and its occurrence in China.

#### ﻿ Melanconis stilbostoma

Taxon classificationFungiDiaporthales﻿Melanconidaceae

(Fr.) Tul. & C. Tul., Select. fung. carpol. (Paris) 2: 115 (1863)

E69CA32E-9D4B-5DBF-BEA1-C92220D4C31B

Fig. 13

##### Description.

***Conidiomata*** acervular, immersed to semi-immersed in the bark, scattered, conical, 850–1200 μm diam., 350–450 μm high. ***Central column*** beneath the disc conical, pale yellow. ***Ectostromatic disc*** dark brown to black, circular to ovoid, 200–400 μm diam. ***Conidiophores*** reduced to conidiogenous cells. ***Conidiogenous cells*** cylindrical, smooth, hyaline to pale brown, producing a conidium at apex, (38–)55–63.5(–69) × 2–3.5(–4) μm. ***Alpha conidia*** brown, aseptate, ovoid, (11.5–)12–13.5(–14.5) × (7–)7.5–8.5(–9) (av. = 12.7 ± 0.7 × 8.1 ± 0.6, n = 50) μm, L/W ratio = 1.4–1.7. ***Beta conidia*** hyaline, aseptate, cylindrical, (7–)7.5–9(–10) × 2–2.5 (av. = 8.4 ± 0.8 × 2.2 ± 0.2, n = 50) μm, L/W ratio = 3.4–4.2.

##### Culture characteristics.

***Colonies*** on PDA flat, spreading, with moderate aerial mycelium and even margin, white to pale brown, reaching 90 mm diam. after 2 weeks at 25 °C.

##### Materials examined.

**China** • Xinjiang Uygur Autonomous Region, Altay Prefecture, Altay City, Xiaodonggou Forest Park, from branches of *Betula pendula*, 6 October 2024, Rong Ma, Caixia Wang & Hailong Lu (XJAU 4035, cultures CFCC 71570, CFCC 71571, CFCC 71572, CFCC 71573, CFCC 71574).

##### Notes.

*Melanconis stilbostoma*, the type species of the genus *Melanconis*, has been reported exclusively on hosts of the genus *Betula* worldwide (Fan et al. 2018b; Jaklitsch and Voglmayr 2020). In China, it is commonly found on branches and twigs of *Betula platyphylla* (Fan et al. 2018b). The morphological characteristics of our collections are consistent with previous descriptions (Fan et al. 2018b; Jaklitsch and Voglmayr 2020). In the present study, this species was identified on branches of *Betula pendula* in XUAR, China, marking its first documented occurrence on this host within the region.

**Figure 13.**
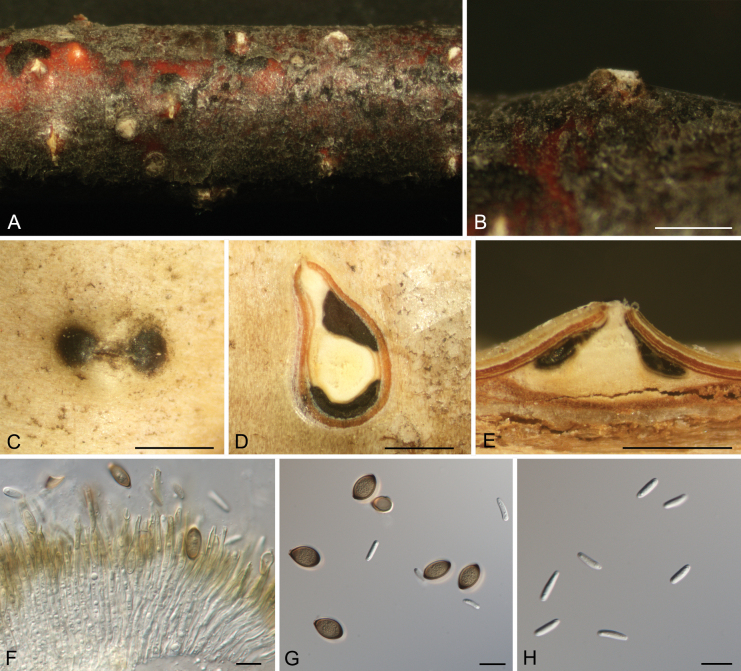
Morphology of *Melanconis stilbostoma* from *Betula pendula*. **A–C.** Habit of conidiomata on branch; **D.** Transverse section through conidiomata; **E.** Longitudinal section through conidiomata; **F.** Conidiophores and conidiogenous cells; **G.** Alpha conidia. **H.** Beta conidia. Scale bars: 500 μm (**B–E**); 10 μm (**F–H**).

## ﻿Discussion

In this study, fungal taxa within the order Diaporthales associated with branch canker diseases of *Betula* were investigated. Through integrated morphological and phylogenetic analyses, eight species were identified. Amongst these, the novel species *Cytospora altayensis* is described herein. Additionally, five existing species *Coryneum lanciforme*, *Cryptosporella betulae*, *Cr. tomentella*, *Cytospora tanaitica* and *Melanconis groenlandica* are reported for the first time in China. *Cytospora sophoriopsis* is recorded for the first time on the host genus *Betula*. Moreover, the asexual morph of *Cryptosporella betulae* was discovered and described for the first time.

Diaporthales is an order of fungi commonly associated with tree hosts, colonising a variety of plant tissues, such as leaves, twigs, branches and stems (Senanayake et al. 2017; Jiang et al. 2020a). Many fungi within this order are responsible for economically and ecologically significant tree diseases. For example, *Cryphonectria parasitica* causes chestnut blight worldwide (Rigling and Prospero 2018), several *Cytospora* species are associated with canker diseases in poplars and willows (Lin et al. 2023, 2024) and *Gnomoniopsis flava* has been identified as the causative agent of nut rot in *Castanopsis carlesii* (Li et al. 2025). Several members of Diaporthales inhabit *Betula* hosts, of which some are pathogens (Table 5).

**Table 5.** A checklist of fungal pathogens inhabiting *Betula* hosts.

Pathogens	Hosts	Distributions	References
*Chapeckia nigrospora*	*Betula* sp.	USA	Fan et al. (2018a)
*Cryptosporella betulae*	*B. pendula*	Austria	Mejía et al. (2008), Mejía et al. (2011)
*Cryptosporella platyphylla*	*B. platyphylla*	China	Fan et al. (2016a)
*Cryptosporella tomentella*	*B. alleghaniensis*	USA	Mejía et al. (2008), Mejía et al. (2011)
*Cytospora leucostoma*	*B. platyphylla*	China	Fan et al. (2018b), Fan et al. (2020)
*Cytospora tanaitica*	*B. pubescens*	Russia	Ariyawansa et al. (2015)
*Diaporthe alleghaniensis*	*B. alleghaniensis*	Canada	Arnold (1967)
*Diaporthe betulae*	*B. platyphylla*	China	Du et al. (2016)
*Diaporthe betulicola*	*B. albosinensis*	China	Du et al. (2016)
*Diaporthe eres*	*B.* sp.	Japan	Kobayashi (1970)
*Discula betulina*	*Betula* sp.	Scotland	Green and MacAskill (2007)
*Diaporthe melanocarpa*	*Betula* sp.	Japan	Kobayashi (1970)
*Fusarium avenaceum*	*Betula* sp.	Scotland	Green (2004), Green and MacAskill (2007)
*Marssonina betulae*	*Betula* sp.	Scotland	Green (2004), Green and MacAskill (2007)
*Melanconis betulae*	*B. albosinensis*	China	Fan et al. (2016b)
*Melanconis groenlandica*	*B. maximowicziana, B. nana, B. papyrifera, Betula* sp.	Denmark, Japan, USA	Lombard et al. (2016), Jaklitsch and Voglmayr (2020)
*Melanconis itoana*	*B.ermanii, B. albosinensis*	Japan, China	Jaklitsch and Voglmayr (2020), Fan et al. (2016a)
*Melanconis larissae*	*Betula* sp.	USA	Jaklitsch and Voglmayr (2020)
*Melanconis stilbostoma*	*B. aetnensis*, *B. papyrifera*, *B. pendula*, *B. platyphylla*	Poland, Italy, Austria, Austria, USA, China	Fan et al. (2016a), Voglmayr et al. (2017), Jaklitsch and Voglmayr (2020)
*Melanconium bicolor*	*Betula* sp.	Scotland	Green and MacAskill (2007)
*Ophiognomonia alni-viridis*	*Betula* sp.	Switzerland, USA	Walker et al. (2012)
*Ophiognomonia hiawathae*	*B. lutea*	USA	Walker et al. (2012)
*Ophiognomonia intermedia*	*B. alba, B. lutea*	UK, USA	Walker et al. (2012)
*Ophiognomonia maximowiczianae*	*B. maximowicziana*	Japan	Walker et al. (2012)
*Ophiognomonia michiganensis*	*B.lenta, B. papyrifera*	USA	Walker et al. (2012)
*Ophiognomonia nana*	*B. nana*	Finland	Walker et al. (2012)
*Ophiognomonia pseudoischnostyla*	*B. pubescens*	Russia	Walker et al. (2012)

Notably, fungal pathogens causing canker diseases on birch trees are of particular concern due to their potential impact on forest health and ecosystem stability (Table 5). As widely distributed pioneer species in both natural and managed forests, birch trees play crucial roles in soil formation, nutrient cycling and providing habitat for various organisms. The emergence of canker pathogens threatens these ecological functions by reducing tree vigour, causing branch dieback and potentially leading to tree mortality. The diversity of Diaporthales species identified in this study highlights the complexity of fungal communities associated with birch cankers and underscores their potential importance in forest decline processes.

*Betula* is an important and widespread tree genus frequently found in natural forests. In this study, eight putative fungal pathogens associated with branch cankers on birch were identified; however, their pathogenicity remains to be confirmed through future experiments.

In XUAR, the diversity of tree species is limited due to the arid climate. Compared to regions, such as Yunnan Province and northwest China, the diversity of Ascomycota, including pathogenic species, is relatively low. This study systematically documents the diversity of Diaporthales associated with birch plants, thereby laying a foundation for future research on the fungal flora in this region.

## Supplementary Material

XML Treatment for Coryneaceae

XML Treatment for Coryneum

XML Treatment for Coryneum lanciforme

XML Treatment for Cytosporaceae

XML Treatment for Cytospora

XML Treatment for Cytospora altayensis

XML Treatment for Cytospora sophoriopsis

XML Treatment for Cytospora tanaitica

XML Treatment for Gnomoniaceae

XML Treatment for Cryptosporella

XML Treatment for Cryptosporella betulae

XML Treatment for Cryptosporella tomentella

XML Treatment for Melanconidaceae

XML Treatment for Melanconis

XML Treatment for Melanconis groenlandica

XML Treatment for Melanconis stilbostoma

## ﻿Additional information

### Use of AI

No use of AI was reported.

### Author ORCIDs

Caixia Wang https://orcid.org/0009-0006-5730-6002

Ning Jiang https://orcid.org/0000-0002-9656-8500

Chuli Liu https://orcid.org/0000-0002-0824-3010

ZiYan Xu https://orcid.org/0009-0008-5044-6461

Hailong Lu https://orcid.org/0009-0001-1583-0846

Rong Ma https://orcid.org/0000-0002-9865-3047

### Data availability

All of the data that support the findings of this study are available in the main text.
